# Reinforcement Learning for decision support in defense and security: A systematic review

**DOI:** 10.1007/s10479-026-07205-x

**Published:** 2026-08-25

**Authors:** Maarten P. D. Schadd, David S. Berman, Carolyn Chen, Mika Cohen, John Dorsch, Alexander Gegov, Maximilian Moll, Oliver Rose, Anna Rösner, Kalle Saastamoinen, Thomas Schiller, Andreas Strand, Lauri Vasankari, Mark Rempel

**Affiliations:** 1 Intelligence & Decision Support, TNO, Oude Waalsdorperweg 63, 2597 AK, The Hague, The Netherlands; 2 Artificial Intelligence Department, Cambridge Consultants, 29, Science Park, Milton, CB4 0DW Cambridge United Kingdom; 3 Center for Theoretical Physics, Queen Mary University of London, Mile End, London, E14NS London United Kingdom; 4 Centre for Operational Research and Analysis, Defence Research and Development Canada, 60 Moodie Dr, Ottawa, K1A 0K2 Ontario Canada; 5 Cyber defence and C2 technology, FOI Swedish Defence Research Agency, Gullfossgatan 6, Stockholm, 164 90 Sweden; 6 Center for Environmental and Technology Ethics, Prague, Institute of Philosophy of the Czech Academy of the Sciences, Celetna 988/38, Prague, 11000 Czechia; 7 Faculty of Philosophy, Philosophy of Science and the Study of Religion, Ludwig-Maximilians-Universität München, Geschwister-Scholl-Platz 1, München, 80539 Germany; 8 School of Computing, University of Portsmouth, Lion Terrace, Portsmouth, PO1 3HE Hampshire United Kingdom; 9 Department of Computer Science, University of the Bundeswehr Munich, Werner-Heisenberg-Weg 39, Neubiberg, 85579 Germany; 10 Department of Military Technology, National Defence University, Kadettikouluntie 7, Helsinki, 00861 Uusimaa Finland; 11 Department of Operations Research and Modeling & Simulation, Army Concepts and Capabilities Development Centre, Colonia Allee 10-20, Cologne, 51067 North-Rhine-Westphalia Germany; 12 Strategic Analyses and Joint Systems, FFI, Instituttvn 20, 2007 Kjeller, Norway

**Keywords:** Reinforcement Learning, Approximate Dynamic Programming, Sequential Decision Making, Defense and Security, Military, Decision Support

## Abstract

Sequential decision making with imperfect knowledge of the environment and adversary lies at the heart of defense and security. As such, contemporary reinforcement learning methods which are well adapted to these sorts of problems have the potential to provide the key tools to support decision makers in a military context. This paper presents a literature review of reinforcement learning for decision support in a defense context with a focus on recent work produced by NATO member states. To aid the reader, we provide the prerequisite background in military decision making; decision support systems in general; reinforcement learning basics; simulation; and explainability. Through a systematic review of the literature and using tools such as UMAP, we construct the research landscape and study the emergent trends and research gaps that are present. This allows us to examine the challenges for exploiting this work in reality and finally, we look to the future and how these challenges can be overcome.

## Introduction

Many decisions are not made in isolation. Instead, they follow a cyclical pattern: decisions are made, new information that was previously uncertain is observed, and further decisions are then based on this new information. This process repeats as additional information becomes available. If how these decisions are made, known as *sequential decision making under uncertainty*, does not consider the relationship between the decisions, then the outcomes achieved may be neither efficient nor effective (Puterman, 1994). Although dynamic programming (Bellman, 1972) provides an elegant framework for finding optimal decision policies for these types of problem, its usefulness is limited in many real-world applications due to the curses of dimensionality and modeling (Bertsekas & Tsitsiklis, 1996; Kuo & Sloan, 2005). In recent years, the fields of Reinforcement Learning (RL) and Approximate Dynamic Programming (ADP)—both of which aim to overcome these curses and fall under the umbrella of Artificial Intelligence (AI)—have produced highly effective decision policies. Within the field of RL, some of the best known examples include policies that: performed as well as human testers playing Atari 2600 games (Mnih et al., 2015); outperformed 99.8% of StarCraft II players at the Grandmaster level (Vinyals et al., 2019); defeated the world champion in the Dota 2 esports game (Berner et al., 2019); and achieved superhuman performance in the games of chess, shogi, and Go (Silver et al., 2018). Likewise, ADP-generated decision policies have been successfully deployed into industry, including policies to schedule drivers in the trucking industry (Powell et al., 2002; Powell, 2007; Simão et al., 2010), plan and manage locomotives (Simão et al., 2008; Powell et al., 2014), and manage high-value spare parts within manufacturing (Simão & Powell, 2009).

**Fig. 1:**
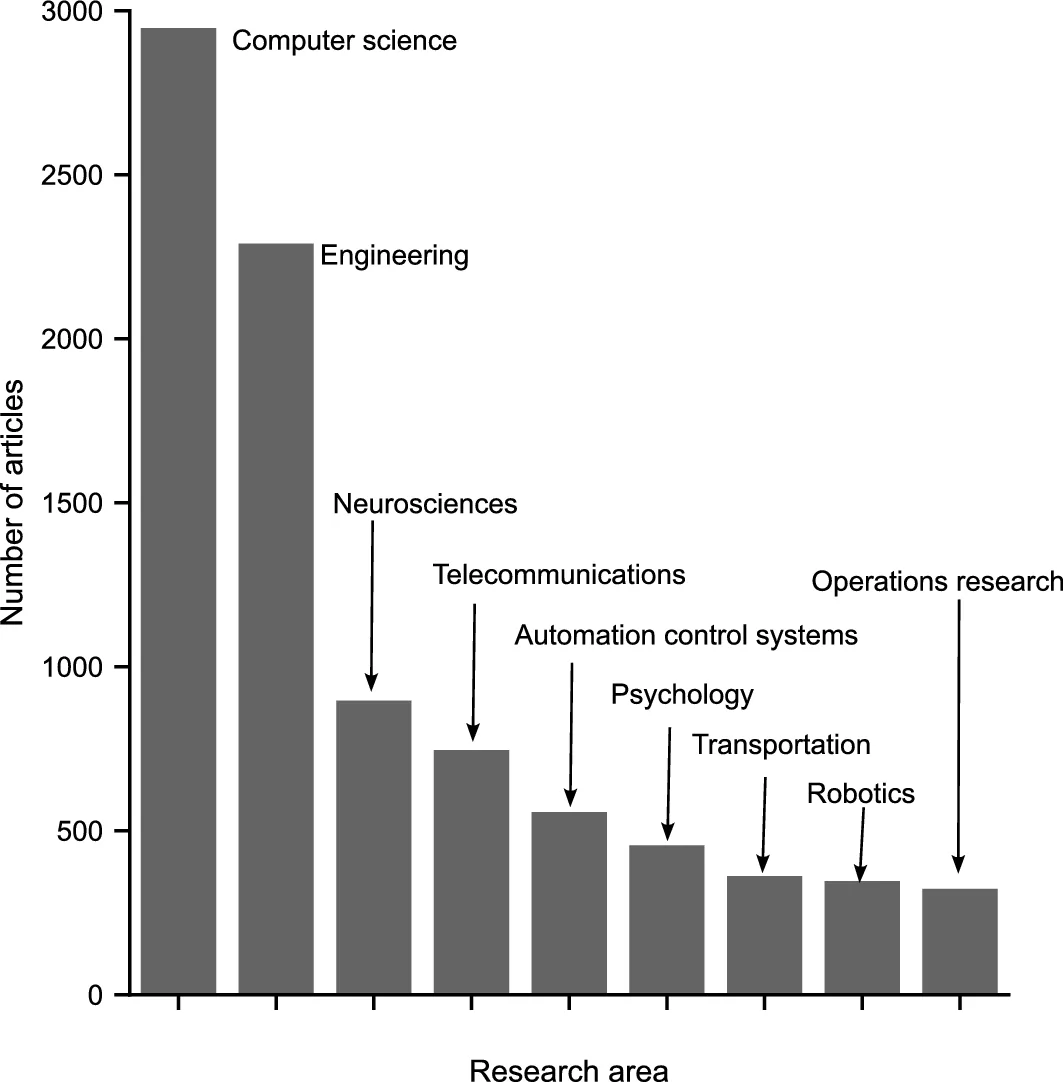
Top ten research areas by number of articles related to reinforcement learning, approximate dynamic programming, decision making, and decision support between 2003 and 20 June 2024. Source: Web of Science using the search pattern (“reinforcement learning” or “approximate dynamic programming”) and (“decision support” or “decision making”) in all searchable fields

**Fig. 2:**
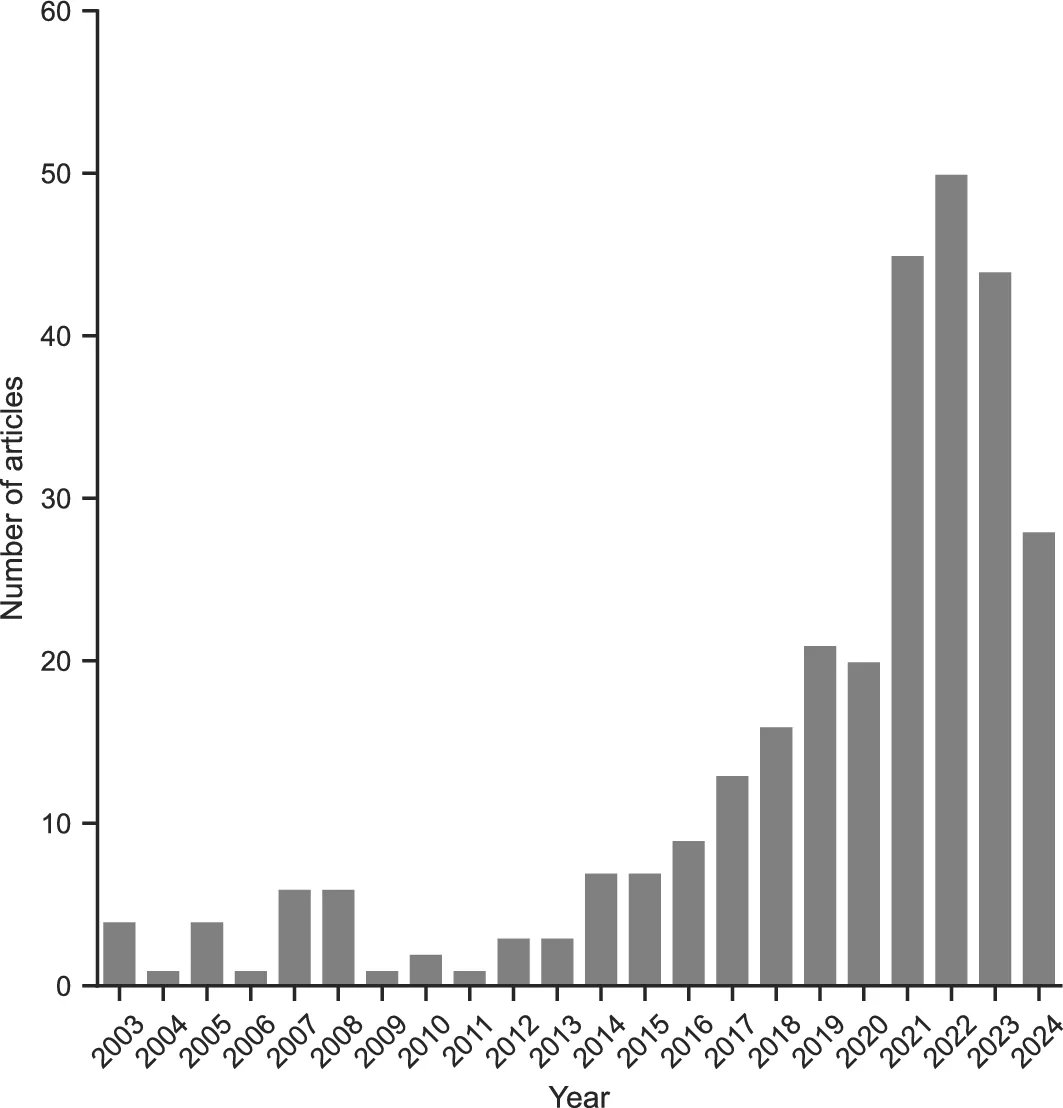
Number of articles related to reinforcement learning, approximate dynamic programming, and decision support between 2003 and 4 June 2024. Source: Web of Science using the search pattern (“reinforcement learning” or “approximate dynamic programming”) and “decision support” in all searchable fields

Beyond these well known examples, research employing RL or ADP—hereafter collectively referred to as RL—has been conducted both in the context of *decision making* (generating a policy that is intended to make a decision without a human-in-the-loop) and *decision support* (generating a policy that is intended to support a human decision maker in some decision-making task) in a wide variety of fields, including computer science, engineering, control systems, robotics, and so on. This is evidenced by Figure 1, which depicts the top ten research areas by number of articles in the last 20+ years that focus on RL and either decision making or decision support. While the overwhelming majority of these articles that are application-based focus on generating decision policies that are intended to be used without a human in the decision-making process, such as maneuvering unmanned aerial vehicles (Huang et al., 2018), autonomous driving (Kiran et al., 2022), and resource allocation within a cloud computing environment (Liu et al., 2017), the popularity of using RL for decision support has increased as depicted in Figure 2, with 292 articles being published between 2003 and June 2024. Similar to those in Figure 1, these publications also cover a wide range of fields, including health care (Liu et al., 2020; Eckardt et al., 2021; Barata et al., 2023), finance (Eilers et al., 2014; Feuerriegel & Prendinger, 2016; Vo et al., 2019), and manufacturing (Gros et al., 2020; He et al., 2021; Papachristou et al., 2021).

Within the context of defense and security, the majority of research on applying RL follows a similar trend and has centered on generating decision policies to be used in autonomous agents, such as those employed in unmanned aerial vehicles, military communication networks, and training simulators. However, recent reviews have highlighted that:

- there is “an increasing role for AI techniques in high-level command and control, strategic planning and decision making” (Moy et al, 2020, p. 18);
- while the majority of ADP-based decision support applications have tended to focus on force employment, a wide variety of potential applications within force development and generation exist (Rempel & Cai, 2021); and
- there is a desire by several nations, such as the United Kingdom, “to apply artificial intelligence techniques to military wargames to allow them to deliver outputs more quickly, cheaply, and/or of higher quality” (Goodman et al, 2020, p. 4).

Collectively these reviews suggest that not only is there a wide array of potential applications to which RL may be used within a defense and security context, but also that there is a desire among nations to explore its potential within the context of decision support. To this end, in 2022 the Science and Technology Organization (STO) within the North Atlantic Treaty Organization (NATO) established a System Analysis and Studies (SAS) Research Task Group (RTG)—SAS-181, *Exploiting reinforcement learning to achieve decision advantage*—with the aim to investigate how RL may be best applied to provide highly effective and reliable decision support within a defense and security context (NATO STO, 2023).^1^ Consisting of eight nations (Canada, Germany, Finland, France, Netherlands, Norway, Sweden, and the United Kingdom), this group’s objectives are to:

- classify, compare, and contrast national efforts that focus on using RL for decision support and identify trends and gaps;
- create a framework whose aim is to guide those designing and developing RL-based decision support systems; and
- identify lessons learned and best practices with respect to both the art and science of using RL in the defense and security domain.

In this article, we address this RTG’s first objective by presenting a review of applications of RL set within a defense and security context. In particular, we focus on articles from those nations participating in the SAS-181 RTG, that are peer-reviewed, and whose primary aim is to provide decision support. With this context, this article’s main contributions are twofold. First, we review 20 decision support applications that span the spectrum of defense planning, force generation, force projection, and force employment that use RL to provide decision support, and describe each using a set of 19 characteristics that cover how the sequential decision problem being addressed was modeled, solved, and the resulting decision policy explained to decision makers. Second, based on this review, we identify trends and gaps in current research, highlight the associated challenges, and offer recommendations to address these challenges and better exploit RL to achieve a decision advantage within a defense and security context.

The remainder of this article is organized as follows. Section 2 provides relevant background information. Section 3 presents the methodology used to conduct this review. Section 4 and Section 5 provide the main contributions of this article. Section 4 reviews the 20 identified applications of RL for decision support within the SAS-181 participating nations, and based on the review Section 5 discusses trends and gaps in the research, challenges, and provides recommendations to address these challenges with the aim of advancing the use of RL for decision support within a defense and security context. Section 6 presents concluding remarks.

## Background

This section provides background information on key concepts to support the remaining sections of this article. As an exhaustive discussion of each topic is beyond the scope of this work, numerous references are provided throughout for the interested reader.

This section is organized as follows. First, an introduction to the main characteristics of decision making in defense and security applications is provided in Subsection 2.1, followed by Subsection 2.2 with a description of Decision Support Systems (DSSs). Next, Subsection 2.3 reviews the topic of sequential decision making under uncertainty, and how RL is used to model such problems and search for good decision policies. Following this, Subsection 2.4 discusses different simulators, and their use within an RL-based DSS. Finally, Subsection 2.5 discusses the concept of explainability and its relation to decision policies learned through applying RL.

A note regarding terminology. In the context of AI, an agent may be defined as “anything that can be viewed as perceiving its **environment** through **sensors** and acting upon the environment through **actuators**”, and that “an agent’s behavior is defined by an **agent function** that maps any given percept sequence to an action” (Russell and Norvig, 2016, pp. 34–35).^2^ The implementation of this function in combination with the architecture on which it is implemented constitutes the structure of an agent. Given the focus of this article is RL-based decision support, we use the term *decision policy* to refer to an agent function, and *RL agent* to refer the combination of a policy, the necessary software components within a DSS to collect data from the environment and recommend actions to a decision maker, and the algorithmic strategy used to learn an optimal, or at least near-optimal, decision policy (Russell and Norvig, 2016, see Fig. 2.15).

### Characteristics of military decision making

In general, two different approaches (or schools) in tactics are present in military decision making and military doctrines.^3^ One is the approach of 19th-century Prussian general and military theorist Carl von Clausewitz (von Clausewitz, 1874). The other is from the Swiss military officer Antoine-Henri Jomini (de Jomini et al., 1854). Clausewitz focused on the psychological dynamics of war, which results in the view that tactics is a form of art. Jomini followed a more probabilistic approach. This school of thought leans tactics more towards a form of science. Today, Western militaries are generally driven by doctrines that lean towards one of these schools, i.e., the United States military is driven by the Jomini approach, whereas the German military is driven by the Clausewitz approach.

Regardless of the school of thought, the conduct of operations, i.e., warfighting, requires commanders to both plan for and make decisions^4^ on multiple levels. These decision-making levels (i.e., generally regarded as strategic, operational, and tactical) vary in terms of scope, referring to scale, impact, and time horizon, and transform the chosen perception of warfare into realizable actions. Strategic decisions relate to long-term, large-scale goals with high but not imminent impact, whereas operational and tactical decisions both relate to smaller scope, impact, and temporal aspects.^5^ Often, the level of decision making is tied to the organizational (hierarchical) level, resulting in high-ranking commanders focusing on strategic rather than tactical decisions.

A range of decision-making styles may be used by commanders to arrive at a given decision. As suggested by Scott and Bruce (1995), these styles may be grouped into four categories: avoidant, intuitive, dependent, and rational. The intuitive style relies on hunches, i.e., subjective experience, insight, and feeling. The dependent style, also known as collaborative, searches for advice and information from others. Rational decision making (Eisenführ et al., 2010) involves a systematic process of defining problems, evaluating alternatives, and utilizing data and logical analysis to find optimal solutions. Any level of decision making may utilize any style, or combination thereof; however, the decision-making style employed often correlates to the decision-making level such that low-level decisions are made intuitively and higher echelons utilize a more systemic, rational approach.

To help commanders employ a systematic, rational approach, an instantiation of the Military Decision-Making Process (MDMP) is often employed (Vowell, 2004). For example, the *United States Army Doctrine Publication 5-0: The Operations Process* (Department of the Army, 2019a) describes the MDMP as the integration of the staff and commander in sequential steps with the aim to produce a plan or order at a tactical or operational level, i.e., suitable for battalion and brigade-level decision making. In this instance, decision support is provided to the commander by: providing insight into the mission; identifying factors that have an effect on the mission, such as intelligence information, environmental analysis, and rules and orders; and developing Course of Actions (COAs) for the commander to evaluate and from which to choose.

**Fig. 3:**
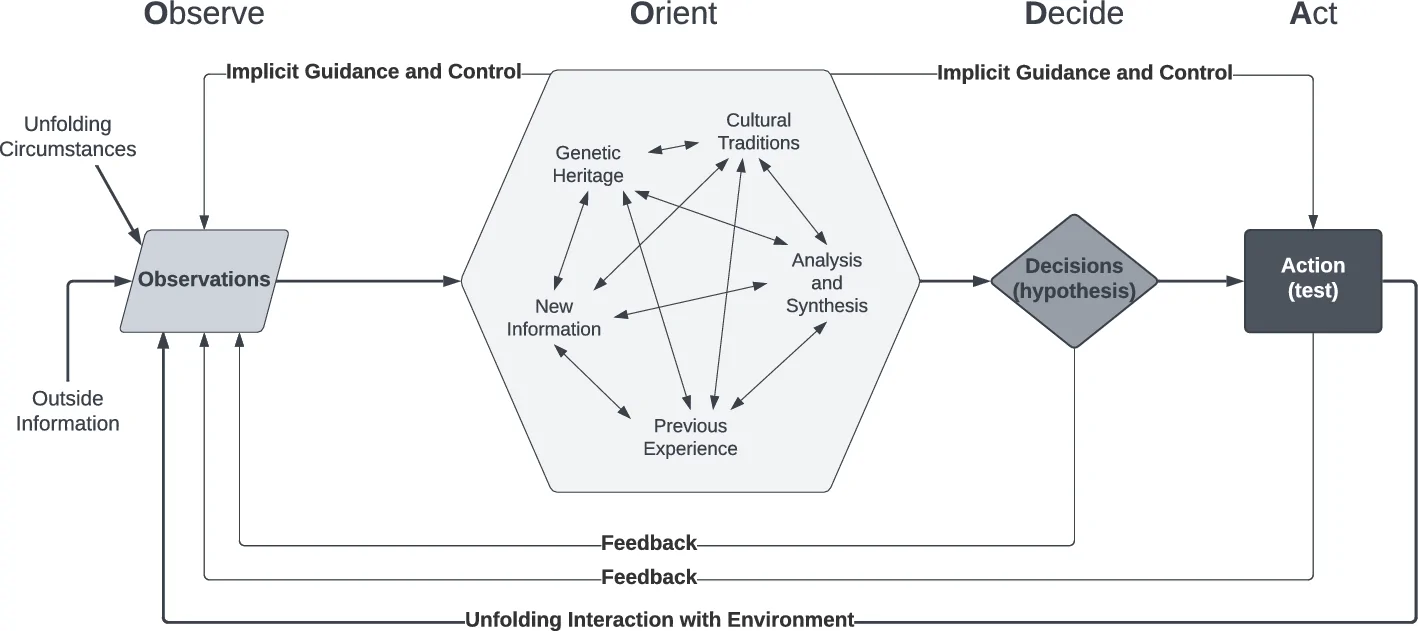
The Observe-Orient-Decide-Act (OODA) loop (Boyd, 2018)

A common framework in which the MDMP is discussed is the Observe - Orient - Decide - Act (OODA)-loop (Boyd, 2018).^6^ This framework, depicted in Figure 3, was originally created to model the decision-making process of a fighter pilot and aims to ensure that all relevant factors, both concrete and obscure, are part of the decision-making process. While Boyd argues the speed of decision making is the key feature to winning battles, i.e., by being more agile and adaptive than the opponent, the framework also highlights the induced bias of the decision maker, which influences their interpretation of the observed situation.

The OODA-loop consists of four stages—*observe*, *orient*, *decide*, *act*—and several feedback paths. The loop begins with collecting *observations* that reflect the state of the unfolding environment and outside information. To decide the suitable action for the perceived situation, the decision maker must *orient* themselves to the observed situation. This orientation is affected by a range of human factors, such as *cultural traditions* and *previous experience*. Orientation leads to a *decision*, which is based on the way observations are processed and interpreted. Lastly, the *action* is the implementation of the decision that ultimately affects the environment, and through feedback updated observations are reflected that were not previously known to the decision maker. Complicating this simplistic perception of the framework are the paths labeled *implicit guidance and control* which showcase situations where the decision maker must react immediately to the available observations, akin to intuitive decision making. Regardless of the pace at which it is executed, the OODA-loop represents a sequential decision-making problem under uncertainty. As these types of problems permeate throughout the defense and security environment (Rempel & Cai, 2021), the OODA-loop is applicable in a wide array of decision problems, including those in defense planning, force generation, force projection, and force employment.

Although not explicitly stated in Figure 3, the observations necessary to make a decision are more often than not only partially observable. This partial observability, commonly referred to as the *fog of war*, may manifest itself as a lack of information, the injection of misinformation, or a delay in receiving the ground truth. Within a military context examples include: spoofing of enemy positions; delayed understanding of the long-term benefits a procurement may provide to a nation’s capabilities; and the uncertain demand for spare parts due to operations. Given such uncertainties are integral to many decision problems with the military, the MDMP and its instantiations may be described as *steps to determine future actions based on incomplete information of the current situation*.

Given the characteristics of the MDMP and OODA-loop—rational decision making, sequential decisions, dynamic information, partial observability of the environment—numerous opportunities exist to provide commanders with decision support. Examples include: wargaming^7^ which may be used as an abstraction of the environment to reveal weak points in a COA and develop mitigation strategies prior to execution (McConnell et al., 2024); a Markov Decision Process (MDP) (Hawkins & Orechovesky, 2024) which may be used to construct a mathematical decision-making model to provide recommendations on which COAs to follow; and the field of AI, including RL, to help commanders and staff develop COAs not previously considered (Schubert et al., 2018). Such approaches benefit from a mathematical model of war, more akin to the Jominian school of thought; however, as long as humans act on and interact with elements on the battlefield, it can be argued that psychological factors cannot be overlooked.

### Decision support systems

Decision support refers to the wide variety of techniques and systems used to assist individuals and organizations in making better and more informed decisions (Power, 2002; Shim et al., 2002). Depending on the application, its aim is to supplement the decision maker’s cognitive abilities, ultimately leading to a more efficient and effective decision-making process. Therefore, decision support falls under the Human-in-the-loop (HITL) paradigm,^8^ which enhances decision making by integrating continuous human involvement with machine intelligence (Holzinger, 2016). This approach is essential in applications requiring human intuition, creativity, and ethical judgment, allowing humans and machines to leverage each other’s strengths and mitigate biases (Bansal et al., 2021). A HITL system that provides decision support is therefore called a DSS, and their implementations range from a simple spreadsheet to a complex software system utilizing various heterogeneous data sources.

**Fig. 4:**
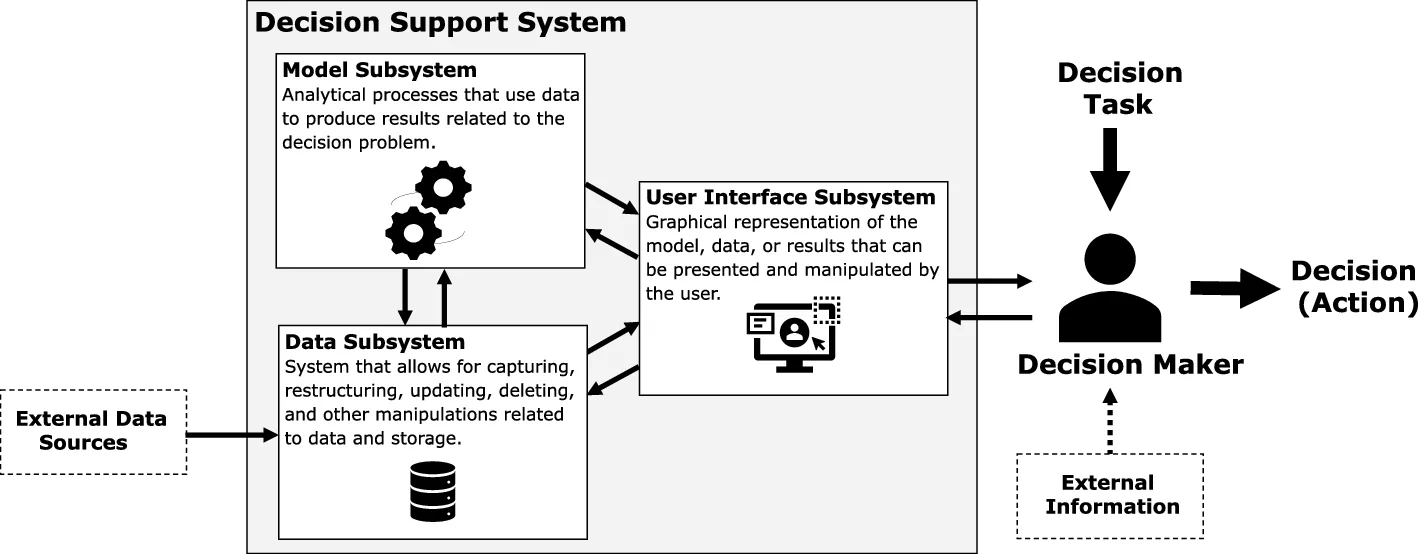
General decision support system architecture

Figure 4 depicts the general architecture of a DSS, differentiating each of the three subsystems—data, model, and user interface—and their internal and external interactions (Sprague, 1980). The inclusion and contributions of each of these components can vary greatly depending on the application of the DSS. This has led to the five classification types of DSS,^9^ each suited to a specific use case (Power, 2002). For example, given a decision problem in which decisions are being made sequentially over time and under uncertainty, a model-driven DSS which focuses on using analytical techniques (such as decision analysis, optimization, and simulation (Power & Sharda, 2007; Bhargava et al., 2007)) within the model subsystem may be the most appropriate.

In the military context, DSSs—which have been described as applied operational research methods for support to the military commander and their staff (Tolk & Kunde, 2000)—have been developed in a broad array of applications, spanning the spectrum from defense planning (Andersson et al., 2015; Rempel & Young, 2017; Chen & Wheaton, 2024) to force employment  (Morrison et al., 1996; Louvieris et al., 2010; Lubkowski et al., 2023). In particular, when considering the MDMP and the OODA-loop depicted in Figure 3, a DSS may be used to support a specific step (such as *Orient* or *Observe*) (Niklasson et al., 2008), or the process as a whole (Blaha, 2018). Regardless, many decision problems being addressed in a defense and security context can be aptly described as sequential decision making under uncertainty, with a range of analytical approaches available to address the decision maker’s needs (Powell, 2022, pp. 8–10). Of these approaches, RL has become a popular choice across several industries in recent years (Liu et al., 2020; Rempel & Cai, 2021; Canese et al., 2021; Gautron et al., 2022).

When employing an RL-based approach, for those problems in which the environment (which generates the observations depicted in Figure 3) is implemented as a simulation, many of the RL components—decision policy, RL agent, environment, etc.—fall under the modeling subsystem. Other components, such as interfaces to external data sources to access exogenous information, fall under the data subsystem. RL and the use of simulation to implement the environment are discussed in more detail in Subsection 2.3 and Subsection 2.4 respectively.

In general, there are two primary approaches to how a learned decision policy generated by an RL-based DSS is intended to be used by a decision maker. First, *direct* RL-based decision support is that in which the policy is used directly to support a decision. The second approach is where the aim is to use RL to learn one or more decision policies that contribute to a higher-level decision. For example, RL may be used to learn how units should maneuver on a battlefield in order to defeat an adversary, while the decision to be supported may be which units are to be grouped together. We label this as *indirect* RL-based decision support.

Lastly, when developing an RL-based DSS, it is important to consider the user interface as this is the only link between the RL agent and decision maker. The design of an RL-based DSS should therefore include considerations for not only how the RL agent will support the decision maker, but also how the decision policy’s recommended actions will be explained. These explanations provide context and meaning to the results which can impact the DSS’s uptake by a decision maker. The importance of explainability is further discussed in Subsection 2.5.

### Reinforcement learning

In this section, we will first provide a brief introduction to RL as a method to solve sequential decision-making problems, and then continue with a more rigorous discussion of this method using the unified framework for sequential decisions. We will motivate our use of this framework and give an outline of its components.

As stated, many decision-making problems in the military are aptly described as sequential decision problems under uncertainty. This leads to a wide range of modeling concepts that may be used to describe such problems, including Markov Decision Processes (Puterman, 1994), Partial Observable Markov Decision Processes (Smallwood & Sondik, 1973), and Powell’s universal modeling framework for sequential decisions (Powell, 2022). In addition, there are several frameworks to solve these types of problems, such as ADP (Powell, 2011), simulation optimization (Osorio & Bierlaire, 2013), and RL (Sutton & Barto, 2018). RL as a solution framework helps us to tackle high-dimensional complex problems, especially when using approaches such as described by Powell (2022). Before we dive into details and apply the above mentioned framework of Powell, we give a brief overview of the basic RL concepts.

RL is a machine learning approach where an agent learns to make decisions by interacting with an environment, often aiming to maximize cumulative rewards. The core components of this approach include the agent, environment, actions, states, rewards, and a policy. The agent observes the state of the environment, takes an action based on its policy, and receives feedback (rewards) that guides its future choices.

**Fig. 5:**
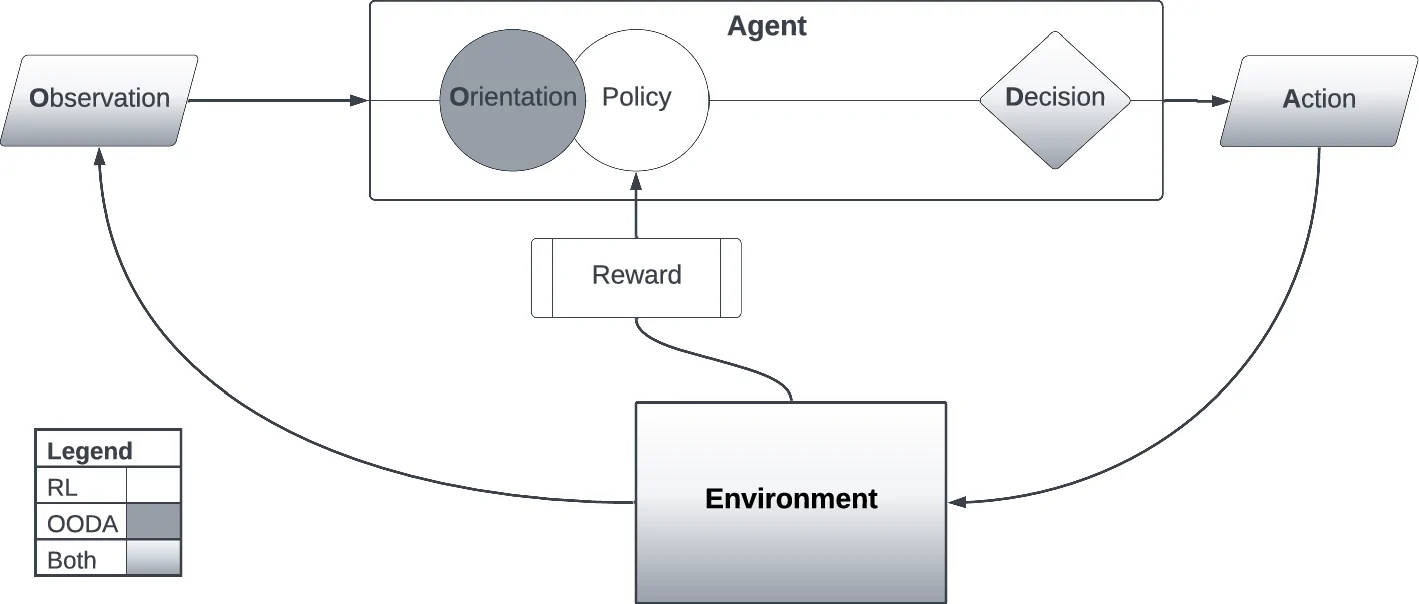
OODA-loop and RL components. Symbols according to ISO 5807 standard

Using RL to learn a policy involves a cycle where the agent observes, acts, receives feedback, and updates its policy. Various methods aid this learning: value-based methods estimate rewards associated with states, policy-based methods directly adjust action selection, and actor-critic methods combine these approaches for enhanced learning. This cycle repeats iteratively until the policy stabilizes and consistently produces actions that maximize rewards. Adjusting hyper-parameters and balancing exploration with exploitation are crucial for efficient learning, allowing the agent to converge to an optimal strategy over time. Thus, the agent learns by exploring the environment and exploiting known information, refining its policy to improve performance through trial and error. Essentially, RL can be perceived as similar to the OODA-loop. The similarities of the cycle from observations to actions and re-orientation are exhibited in Figure 5, where gray indicates resemblance to the OODA-loop, white highlights RL components, and gradients represent both.

A central challenge in RL is the exploration–exploitation trade-off, in which an agent must balance learning about uncertain state–action outcomes with exploiting existing knowledge to maximize reward. This problem has been studied extensively in the adaptive control and MDP literature, where asymptotically optimal adaptive policies are designed to minimize long-term regret.^10^ In particular, Burnetas and Katehakis (1997) provide foundational results on adaptive policies for MDPs that achieve optimal reward growth through principled exploration. Recent work by Cowan et al. (2020) further shows that structural properties of the underlying MDP, such as state connectivity and transition uncertainty, critically affect policy complexity and convergence rates.

**Fig. 6:**
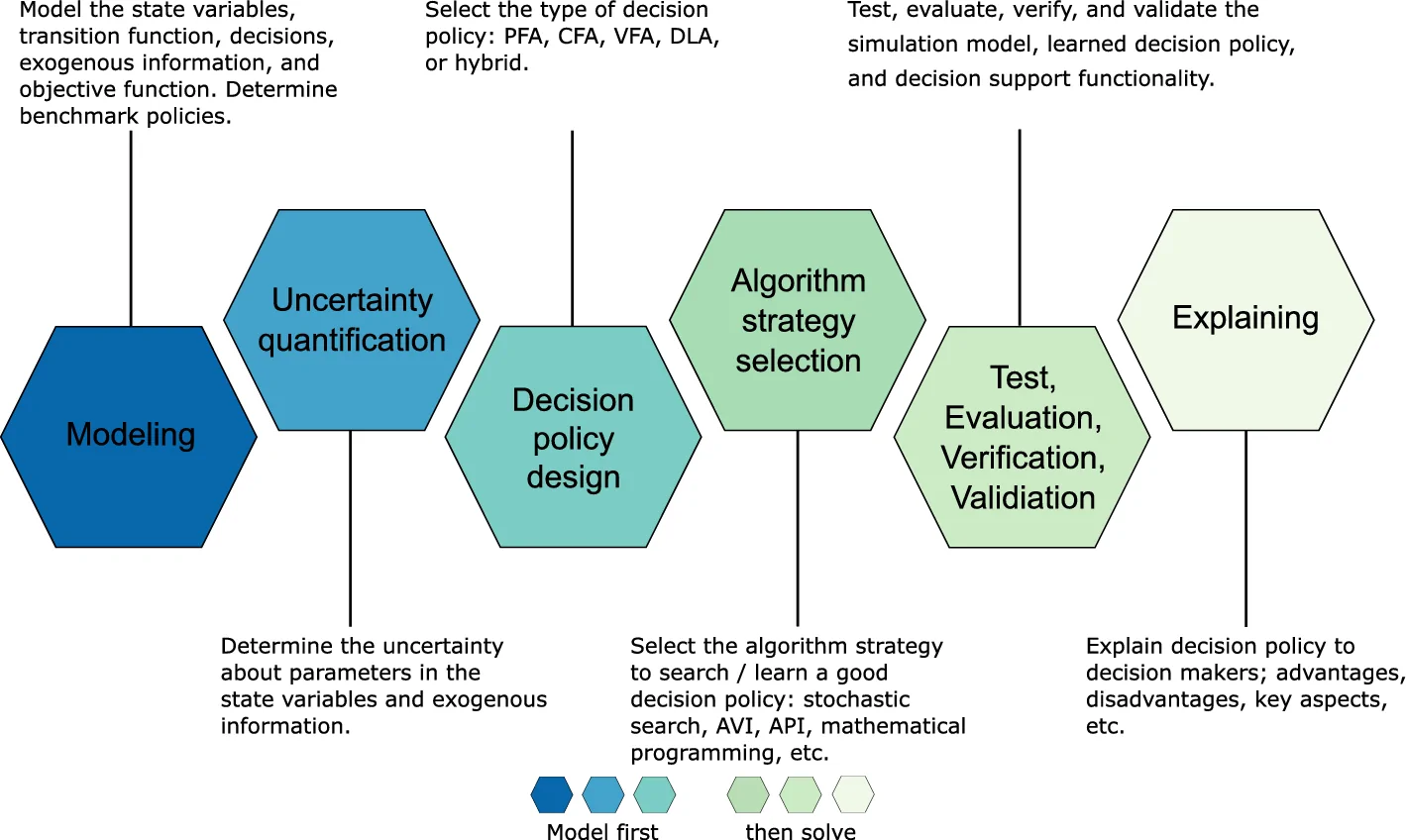
Extension of Powell’s unified framework for sequential decisions. The original framework is extended so that the evaluation step includes: the test, evaluation, verification, and validation of the simulation model, learned decision policy, and decision support functionality; and a step focused on the explainability of the decision policy

As mentioned, RL is one of many approaches to solve a sequential decision problem where these approaches use “roughly eight notational systems, and an overlapping set of algorithmic strategies” (Powell, 2022, p. 8). Powell’s *universal modeling framework for sequential decisions* aims to provide a consistent modeling language for sequential decisions, thus providing the flexibility to employ methods from multiple communities to determine optimal, or at least near-optimal, decision policies (Powell, 2022, Ch. 9 through Ch. 11). This modeling framework, which consists of three steps—modeling, uncertainty quantification, decision policy design—is a component of Powell’s larger *unified framework for sequential decisions*. This larger unified framework, which takes a *model first, then solve* approach, includes the three aforementioned modeling steps, plus two *solving* steps, namely algorithm strategy selection and evaluation. For the purposes of this review we extend Powell’s original unified framework. First, we explicitly incorporate testing, verification, and validation in the evaluation step as both of these are important activities for any DSS used in a military context. Second, we add a sixth step focused on explainability of the learned decision policy to decision makers. These six steps are depicted in Figure 6, where each hexagon represents a single step. Each of the six steps are briefly described in the remainder of this section. For the interested reader, three recent examples of using Powell’s universal modeling framework for sequential decisions in a defense context are found in Amyot-Bourgeois and Rempel (2023), Rempel (2024), and Chen et al. (2025).

#### Modeling

Within the universal modeling framework, a sequential decision problem is described using five elements (Powell, 2022, Ch. 9):

- **State variables** $S_t$: Information that is necessary and sufficient to compute the contribution function, decision policy (including its constraints), and transition function. For example, $S_t$ may include a set of candidate projects to invest in at decision epoch *t* and the available budget.
- **Decision variables** $x_t$: The decisions that are to be made and controlled by the decision maker. These are subject to constraints $x_t \in \mathcal {X}_t$ and are made via a policy $X^\pi (S_t)$. For example, $x_t$ may include the decision to invest in project A and not in project B at decision epoch *t*, or a request to transfer available budget between fiscal years.
- **Exogenous information** $W_{t+1}$: Information that is learnt after a decision is made, i.e., it is uncertain at the time a decision is made, and comes from outside the system that is being modeled. For example, $W_{t + 1}$ may include new candidate projects that become available in decision epoch t+1 or changes to the available budget in decision epoch t+1.
- **Transition function** $S^M(S_t, x_t, W_{t+1})$: A function that represents the dynamics of the system being modeled and that updates the elements of the state variable to generate $S_{t + 1}$. For example, the function may compute changes to the available budgets in t+1,t+2,… given how successful earlier investments of decision epoch *t* were, or changes in future budgets given the exogenous information.
- **Objective function**: Given a contribution (or reward) $C(S_t, X^\pi (S_t))$ that is received at each decision epoch *t*,^11^ the most common way to write the objective function is as a cumulative reward given as^12^

1 $$\begin{aligned} \max _{\pi \in \Pi } {{\,\mathrm{\mathbb {E}}\,}}\left( \sum _{t = 0}^{T - 1} \gamma ^t C(S_t, X^\pi (S_t)) | S_0 \right) , \end{aligned}$$

where $S_0$ is the initial state and the expectation is taken over all relevant sources of uncertainty. The formulation follows the general modeling framework of Powell (2022), which allows for both finite-horizon and infinite-horizon problems. In finite-horizon settings, such as time-bounded military missions or operations, the discount factor γ is typically set to 1, yielding an undiscounted cumulative reward. When γ<1, the formulation corresponds either to an infinite-horizon problem or to a modeling choice that emphasizes earlier rewards. The max operator simply means to find the policy π in the family of policies Π that results in the best expected outcome. Alternative objective functions, including terminal reward or regret-based formulations, may also be employed (Bertsekas & Tsitsiklis, 2000; Jaksch et al., 2010).

Due to the need to cope with multiple decision makers in many real-world problems, the basic RL setting (which is reflected in the universal modeling framework) has been extended to Multi-Agent Reinforcement Learning (MARL) where more than one agent can learn simultaneously. This includes both competitive (Chen et al., 2024) as well cooperative settings (Low & Zhou, 2025). In both cases, all agents interact with the same environment.^13^ Importantly, the joint state-action space grows exponentially with the number of agents *N*, i.e., $|\mathcal {S}| \times |\mathcal {X}|^N$, which is a primary computational bottleneck in many multi-agent applications. As such, the basic setting, and hence the modeling framework described above, needs to be extended to (potentially partially-observable) stochastic games (Ning & Xie, 2024).

In addition to multiple decision makers, many real-world problems also include multiple objectives. To address these situations, the field of Multi-objective reinforcement learning (MORL) spans various settings of relevance to (military) decision making. In its most basic setting, it responds to the need to balance competing goals, like mission success, civilian risk, logistics costs, and others. Rather than considering simple weighted sums, such situations can be modeled with vector-valued rewards (Van Moffaert & Nowé,^2014^) and Pareto-optimal policy sets (Basaklar et al., 2022). In particularly high-stakes operations the extension to distributional MORL targets risk-aware Pareto optimality over full return distributions (Cai et al., 2023). For the consideration of safety and constraints (Achiam et al., 2017), recent approaches (Kotecha & Del Rio Chanona, 2025) and methods integrate chance constraints (Pfrommer et al., 2022), Conditional Value at Risk (CVaR) (Ying et al., 2022), and constrained MDP formulations so agents respect hard rules of engagement and safety envelopes while optimizing multiple objectives (Ren et al., 2023). Cutting-edge research (Liu et al., 2024) also explores constrained multi-objective formulations that jointly satisfy strict safety while balancing mission metrics.

Given these developments within the RL community to address these more complex problems, the universal modeling framework for sequential decisions has been extended to address multi-agent, multi-objective problems. In regard to multiple agents, a multi-agent extension has been developed that “can handle uncertainty in the state of the world, uncertainty in the policies of other agents, and uncertainty in the dynamic stochastic processes” (Thul, 2022, p. 3). For further details, see Ch. 20 in Powell’s book *Reinforcement learning and stochastic optimization* (Powell, 2022).

#### Uncertainty quantification

In order to find an effective decision policy $$X^\pi (S_t)$$, accurate identification and modeling of the uncertainty within the problem at hand is required. Under the assumption that the initial state $$S_0$$ is given, which may include assumptions about parameters of probability distributions used when generating $$W_{t + 1} $$ and $$S_{t + 1}$$, Powell identifies several forms of uncertainty that may be resident in a sequential decision problem. These are briefly summarized in Table 1.

As an example within a defense context, Birkemo (2013) identified several uncertainties that exist within the Norwegian defense planning process, including: political (strategic shocks such as the end of the Cold War, types of scenarios considered), exogenous (future budgets, adversarial uncertainty in terms of enemy goals), goal (level of ambition in scenarios), and so on.

**Table 1:** Summary of forms of uncertainty within a sequential decision problem. See (Powell, 2022, Ch. 10) for a complete description

Uncertainty form	Description
Observational errors	Arises when the state variables are not known, cannot be observed directly, or cannot be measured accurately.
Exogenous	Arises within the new information $W_{t + 1}$ from outside the system under study. Includes: uncertainty that varies between two consecutive time-steps (aleatoric); uncertainty that occurs over long time scales (systematic); uncertainty regarding the types or parameters of distributions; and if an adversary has selected $W_{t + 1}$, then uncertainty in its behavior.
Prognostic	Reflects deviations between forecasted value and their actual values.
Inferential	Arises when observations, which may be noisy, are used to draw inferences about other parameters, of which there is then uncertainty. May also be referred to as diagnostic uncertainty.
Experimental variability	Differences between results obtained under the same experimental conditions, such as in laboratory experiments, numerical simulations, and field testing.
Model	Arises as errors in estimated parameters of parametric models, or in the structure of the model itself. May become apparent in the contribution function, transition function, or constraints.
Transitional	Arises when exogenous noise affects the transition from $S_t$ to $S_{t + 1}$, although it is not accounted for in the modeled transition function itself.
Control / implementation	Reflects errors made when implementing a decision $x_t$.
Communication errors and biases	Arises in multi-agent systems when two or more agents communicate with each other. Includes both errors in the communicated information and biases.
Algorithmic instability	Uncertainty that results from the algorithm used to solve the problem, such as when the algorithm uses Monte Carlo sampling, is sensitive to small changes in input data, the algorithm is a parallel implementation, etc.
Goal	Arises in problems with multiple competing objectives and the decision maker’s preference between those objectives is uncertain, or when some objectives are not articulated.
Political / regulatory	Arises in problems that concern long-term planning. May include changes in government, laws, geo-political situation, etc.

Following the identification of relevant uncertainties is the need to generate sequences of outcomes that represent samples of their observations. These samples may be drawn from probability distributions, historical data, directly from a physical system or process, large computer models, or a set of outcomes that may be rare but for which must be planned. In addition, the correlations between samples, such as those over time, space, or with respect to characteristics, may need to be taken into account. For an example, see Cobb (2014) for modeling uncertainties in military supply-chain-management decisions.

#### Decision policy design

There are four classes of decision policies (Powell, 2011, 2022): myopic, and its extension known as myopic Cost Function Approximation (CFA); Policy Function Approximation (PFA); Value Function Approximation (VFA); and Direct Lookahead Approximation (DLA).^14^ In addition, hybrid policies may be created by mixing policies from two or more of these categories. Table 2 lists the policy classes, an example policy for each, and a description of the policy class.

**Table 2:** Summary of decision policies. See (Powell, 2011, Ch. 6) and (Powell, 2022, Ch. 11) for a complete description

Class	Example policy and class description
CFA	$X^\pi (S_t) = \mathop {\mathrm {arg\,max}}\limits \limits _{x_t \in \mathcal {X}_t} C(S_t, x_t)$
	Makes decisions based on near-term rewards without regard to long-term impacts. These policies may be parameterized by a vector θ, include a range of problem-specific constraints, or correction terms.
PFA	$X^\pi (S_t | \theta ) = \theta _0 + \theta _1 \phi _1( S_t)$
	Returns a decision $x_t$ directly based on the state $S_t$ without solving an optimization problem. May take the form of a lookup table, e.g., if military airlift is not available, then use contracted airlift; a parametric model; or a nonparametric model.
VFA	$X^\pi (S_t | \theta ) = \mathop {\mathrm {arg\,max}}\limits \limits _{x_t \in \mathcal {X}_t} (C(S_t, x_t) + \gamma \sum _{f \in \mathcal {F}} \theta _f \phi _f(S^x_t))$
	Explicitly accounts for the downstream impact of a decision through approximating its value around the post-decision state variable $S^x_t$. Approximation may take the form of a lookup table, parametric, or nonparametric approximation.
DLA	$X^\pi (S_t | \theta ) = \mathop {\mathrm {arg\,max}}\limits \limits _{\tilde{x}_{tt},\ldots ,\tilde{x}_{t,t+H}} \sum _{t^\prime = t}^{t + H} C(\tilde{S}_{tt^\prime }, \tilde{x}_{tt^\prime })$
	Makes a decision at t=0 by optimizing decisions over a finite horizon from *t* to t+H. When the number of possible actions and outcomes over a short time horizon are small, an approach based on tree search, using either complete enumeration, Monte Carlo sampling of outcomes, or employing a roll-out heuristic to evaluate what may happen once reaching a state, is feasible (Powell, 2011, pp. 225–227). When the problem involves a vector of decisions, a deterministic or stochastic DLA based on mathematical programming may be used (deterministic example shown). In the deterministic case, point forecasts of future exogenous information available at time *t* are used to design the policy, such as Powell (2014, p. 121–122). In the stochastic case, point estimates are replaced by sample realizations.

The choice of policy for a given problem depends on many factors, including the characteristics of the problem itself, available computational time, and the complexity of the algorithm used to seek an optimal policy. For example, PFAs may be a good choice when the structure of the policy is well understood; CFAs may work well when a reasonable deterministic optimization model that is a good approximation is available; VFAs are appropriate when the impact of a decision made today on the future is important and can be captured in a well-defined function, such as a linear or concave function; and DLAs may be a good choice when planning must be done over a horizon in order to make a decision now. For a discussion on choosing a policy class, see Sec. 11.10 of Powell’s recent book (Powell, 2022).

#### Algorithm strategy selection

A wide variety of algorithmic strategies exist that may be used to search for a good decision policy, such as Q-learning (Watkins & Dayan, 1992), Proximal Policy Optimization (PPO) (Schulman et al., 2017), and Monte Carlo Tree Search (MCTS) (Vodopivec et al., 2017). For many of these algorithms, variations exist for settings beyond standard RL, such as MAPPO, which extends the single agent PPO to the multi-agent setting (Yu et al., 2022) for CTDE, or preference-conditioned policy families (Reymond et al., 2022) and multi-objective PPO variants (Xu et al., 2023) for MORL.

Unfortunately, choosing a strategy for a particular problem is not straightforward. While research exists that aims to provide guidance (Almahamid & Grolinger, 2021; Schröder et al., 2024; Bongratz et al., 2024), many factors must be considered, such as whether: the learned policy will be static or adaptive (Burnetas & Katehakis, 1997; Cowan et al., 2020); the learning will be done offline or online (Levine et al., 2020; Sujit et al., 2023); and the class of decision policy itself (Powell, 2011, 2022). In the remainder of this section, we focus on how the choice of decision policy impacts the algorithm strategy selected. An in-depth discussion of each strategy is beyond the scope of this work; for a deeper appreciation of these algorithms see previously cited works by Powell and the references provided below.

When a PFA is characterized by a vector θ of tunable parameters, searching for a good policy means searching for the best vector θ. This may be done by a range of stochastic search algorithms, such as simultaneous perturbation stochastic approximation (Spall, 2003; Bhatnagar et al., 2013), genetic algorithms (Spall, 2003), or an exact derivative if possible.

A myopic CFA is in essence an optimization model, that may or may not be parameterized by a vector θ of tunable parameters. In the former case, the parameters may be tuned via stochastic search while optimization software is used to determine a decision for a given parameter vector; whereas in the latter case, the decision function may be solved via appropriate optimization software alone.

When using a VFA-based decision policy, searching for a good policy is akin to tuning the approximation’s parameter vector θ. Common strategies employed are Approximate Value Iteration (AVI), Approximate Policy Iteration (API), and Q-learning (QL). These algorithms are widely used within RL and ADP; however, implementations tend to be problem specific—see (Powell, 2011, Ch. 10) for several examples and Bertsekas (2011) for an overview of API. Factors affecting an algorithm’s design include if the problem studied has a finite or infinite horizon, the type of value function approximation chosen, and how the approximation’s parameter vector θ is learned.

Lastly, when using a DLA-based decision policy, this in essence is a mathematical program—deterministic in the case of point forecasts, and stochastic when point estimates are replaced by sample realizations. In the latter situation, two-stage or multistage stochastic programming are core approaches (Birge & Louveaux, 2011), whereas in the former algorithms implemented in commercial optimization solvers are most appropriate.

#### Test, evaluation, verification and validation

An important step is the execution of Test, Evaluation, Verification, and Validation (TEVV) activities. TEVV contain various activities that cover five dimensions and seven lifecycle stages (National Institute of Standards and Technology, 2023, p. 11). All these activities are relevant for RL-based DSSs, but in this section we focus on the AI Model and Task & Output dimensions. Following the general DSS architecture, we discuss TEVV activities for the simulation model (i.e., Model Subsystem with data from the Data Subsystem of Figure 4), the learned decision policy (i.e., Model Subsystem of Figure 4), and the decision support functionality (i.e., User Interface Subsystem of Figure 4). TEVV activities have to be performed in close interaction between developer and user.

As described in more detail in Subsection 2.4, the *simulation model* is a core part of the system which is used for training and evaluation of the decision policy. For simulation models, Sargent (2010, 2020) and Sargent and Balci (2017) provide a good overview on verification and validation methods. Verification is typically done by means of checklists with which the existence of a particular simulation-model characteristic is assessed and then documented (Rakitin, 2001).

For the *decision policy*, TEVV activities address the question whether the decision policy makes sensible decisions. The verification is usually done by assessing if the policy is built according to given specifications, and Powell’s framework provides a guideline of the required components. The main validation approach is to compare the expected objective values generated using the decision policies under study to selected benchmark policies. These benchmark policies may include: optimal policies, such as an optimal policy via dynamic programming or an optimal deterministic version of the problem (Powell, 2009); currently implemented policies, such as rule-based policies (PFAs); policies that have been learned in previous development iterations; or a qualitative evaluation against the policy inside a decision maker’s mind. The aim of this comparison is to determine if a learned policy outperforms the selected benchmark(s), and if so by how much.

When TEVV activities point out shortcomings of the learned decision policy, the problem may not only be in the policy itself, but may also originate from the simulation model. Any problem in the simulation model has impact on the learned decision policy. The decision policy may have learned to exploit bugs in the simulator or learned undesired behavior (Baker et al., 2019). Therefore TEVV activities for the decision policy may trigger new requirements and TEVV iterations for the simulation model.^15^

Finally, the *decision support functionality* should be addressed by TEVV activities. After integration of the verified and validated policy into the DSS, its performance has to be tested with respect to the goals of the decision maker. The DSS should be valuable in diverse, realistic, and potentially unseen conditions, leading to requirements for robustness and generalizability. An evaluation may be performed to gain insights into algorithmic performance and application-specific metrics such as decision quality in combat scenarios or mission success rates (Bunch et al., 2024; Lalitha et al., 2023). It also has to be assessed whether the RL agent creates useful results in a timely manner. The performance evaluation of the simulation model relates to the training phase. The execution of large (military) simulation models is notoriously slow. During the evaluation, it has to be assessed whether the existing computer environment is potent enough to complete the training of the decision policy fast enough.

These TEVV activities support the model developer in producing appropriate model artifacts and increase trustworthiness to their users. They do not guarantee perfect simulation models, decision policies or DSSs, but they improve the quality of the aforementioned by establishing best practices.

#### Explaining

In military decision making, explaining the decision policy that is learned via RL is essential for transparency, trust, and accountability. Understanding how the policy determines which actions to recommend enables commanders to validate its actions, ensuring they align with mission objectives and ethical responsibilities. This clarity allows operators to adapt strategies to changing battlefield conditions, maintain compliance with legal and policy standards, and promote effective human-machine collaboration. Explainability also aids in debugging and continuous improvement, helping refine the policy’s performance in complex, high-stakes environments where lives and mission success are on the line. Further details can be found in Subsection 2.5.

### Simulators

A simulator is a device, software, or system that replicates or mimics the behavior of a real-world process, system, or environment for various purposes such as training, decision support, research, testing, or entertainment. Simulators are used in a wide range of fields, including aviation, medicine, military, engineering, and video gaming. The open archive of the Winter Simulation Conference (2024) provides an overview on the theory and applications of discrete-event computer simulation starting from 1968. In this review, we focus on simulation software tools for military decision making; however, we will not dive into detail with respect to military simulators as the NATO STO (2024b) offers vast materials. In addition, there is a considerably body of literature on that topic. A reasonable starting point are the books (co-)authored by Andreas Tolk, such as Tolk (2012) or Ören et al. (2023).

An overview of military simulation tools can be found in Table 1 of Van Oijen and Toubman (2021). Although both civilian and military simulation tools are used to support decision making, military tools are mainly used by decision makers, and mostly not by trained operations-research personnel. Thus, the scope of most military simulation tools lies more in visualization and immersion, focusing on high realism rather than on fast simulation speeds and experimental design capabilities.

In the DSS structure (cf. Subsection 2.2), the simulator is part of the model subsystem. More specifically, it is an application which is used to run an abstract representation of the system of interest, the simulation model. In Powell’s terms, the simulation model can be a model of the transition function, of exogenous information, or the reward function. The scope and the level of detail of the model has to match the level of abstraction of the decision policy. For military simulation, building appropriate simulation models can be difficult due to a lack of available data, in particular about stochastic parameters like availabilities of critical resources such as weapon systems. An in-depth discussion of model fidelity aspects for military decision making systems can be found in Weyland et al. (2024).

Next, we describe how simulators and DSS components interact. In general, the current approach is to take an existing RL software framework (mainly implemented in Python) to implement an RL agent and integrate a simulation engine via interfaces. A typical implementation is to make the simulator read/write the relevant data of the decision policy. This concept is used both in the training and deployment of the learned decision policy. This is difficult for commercial simulators as this data exchange is usually not provided per se, but has to be implemented by the tool vendor. This is one of the main reasons that currently, the majority of simulation-based RL is implemented with bespoke simulators.

Simulators can be used both during the training phase, where the aim is to learn a good decision, and the deployment phase. During the training phase, the RL agent controls the simulation runs. It sets the initial state of the simulator, determines (an) action(s) to be performed, starts the simulator, waits for evaluations of the reward functions during or after the simulation run, and uses the simulated environment data obtained to update the RL agents’s parameters (cf. Section 2.3). As soon as a stopping criterion is met, these iterations come to an end with a learned policy. During the deployment phase, the simulator calls the learned policy with state data to receive the respective recommended action to be provided to the decision maker.

Due to the intensive exchange of information between simulator and RL agent, this can be the source of considerable speed losses, mainly during the training phase, lesser during the deployment phase. Typically, most of the training time is consumed by the simulation-based computation of the transition function, exogenous information, and reward function. Therefore, running as many simulation runs as possible in parallel can be very beneficial.

Another prominent issue is that the simulation model has to match the scope and the level of detail of the data needs of the RL agent. There can be both lack and abundance of simulation data. If data to learn a good decision policy is missing, the simulation model will have to be extended (e.g., more components and/or more relationships between components and/or higher level of detail in structure and/or behavior). If the model provides more detailed data than is required, then data has to be aggregated. This issue mainly relates to the set of state variables. It is not always clear how to aggregate them into super-sets, characteristics, or features.

Among commercial military simulators which have a potential to be used in conjunction with RL, such as BSI MACE and ARMOR (Battlespace Simulations Inc, 2024), JTLS GO (Valkyrie Enterprises, 2024), PAXSEM (Kallfass & Schlaak, 2012), or Aqlab (Avioniq, 2024), only for the military sibling of the computer game Command: Modern Operations (CMO) (Matrix Games, 2024) material about simulation-based RL can be found, such as PyCMO (Hua, 2023). Apart from commercial military simulators, there is a number of bespoke discrete-event simulation tools which are used in the military, such as battlefield simulators as in Möbius et al. (2022, 2023b).

An overview about simulation-based RL in the civilian domain from 2022 can be found in Belsare et al. (2022). At the time of writing, from the major simulation tool vendors, only Flexsim (2024) and AnyLogic (The AnyLogic Company, 2024) mention RL support in their online material. Most publications about the combinations of simulators and RL are about academic implementation experiments, from which we conclude that most of the tool vendors seem not to be ready yet for integrating RL and academics tried to find ways to overcome this situation with little or no support from the vendors. Examples of this approach are Siemens Plant Simulation (Kemmerling et al., 2021) or Simio (Greasley, 2020). More support can be found for the open source simulation package SimPy (Simpy, 2024), e.g., in factory optimization (Singh, 2024).

### Explainability in RL-based decision support systems

Crucially, the effective integration of DSSs into human decision making requires its suggested decisions be transparent and understandable to the decision maker with little to no expertise in machine learning, a goal pursued by Explainable Artificial Intelligence (XAI) in general and Explainable Reinforcement Learning (XRL) in particular. But success has been limited because XAI is threatened by the “inmates running the asylum problem” (Miller et al., 2017, p. 1), wherein explanations are designed for developers (the inmates) rather than for decision makers, who, unfamiliar with machine learning methods, do not fully understand the explanations (for more, see Dorsch and Moll (2024)).

For this reason, a growing consensus is emerging for distinguishing XAI from Interpretable AI (IAI). IAI is about, “understanding how the model makes decisions, based on a holistic view of its features and each of the learned components such as weights, other parameters, and structures” (Molnar, 2022, §3.32). XAI goes beyond interpretability by generating deployer-understandable insights into how DSSs arrive at specific outcomes. Furthermore, Doshi-Velez and Kim (2017) argue that explanations ought to be validated by empirical studies with human participants, perhaps interacting with simplified models. To clarify this distinction Table 3 lists several approaches for XRL that fall under IAI and XAI. Overall, many approaches for interpretable and explainable RL have been proposed, and several review articles have been published, which provide an overview of the ongoing research (Bekkemoen, 2024; Saulières, 2025; Milani et al., 2024; Qing et al., 2022). These survey papers use different taxonomies to differentiate between interpretability and explainability. While a detailed comparison of all current XRL approaches is beyond the scope of this article, these surveys extend the examples presented in Table 3. In addition, several researchers have proposed ideas for explainability checklists for auditing or score cards ( Winikoff et al. (2025); Sokol and Flach (2020); Langer et al. (2021)). However, these approaches differ in scope and methodology. They are often tailored to specific use cases (for example, the type of explainer employed or the nature of the data), which makes it difficult to define a universal checklist for explainability.

**Table 3:** Examples of interpretable AI (IAI) and explainable AI (XAI) approaches for RL

Category	Example	Definition
Interpretable AI	Decision Tree RL (LMUT, VIPER) Bastani et al. (2018)	Tree-based RL policies are inherently interpretable by mapping states to actions via transparent rules.
	Formula-based Q-functions Inala et al. (2020)	Policies approximated with closed-form mathematical expressions.
	Attention-based interpretability through saliency maps Madumal et al. (2020)	Highlights important state features or attention regions to aid interpretability.
Explainable AI	Counterfactual Explanations Gajcin (2020)	Explains “why action X instead of Y” by showing minimal state/action changes that alter decisions.
	Instruction-based explanations Fukuchi et al. (2017)	Agent generates instructions to explain its chosen behavior.

**Fig. 7:**
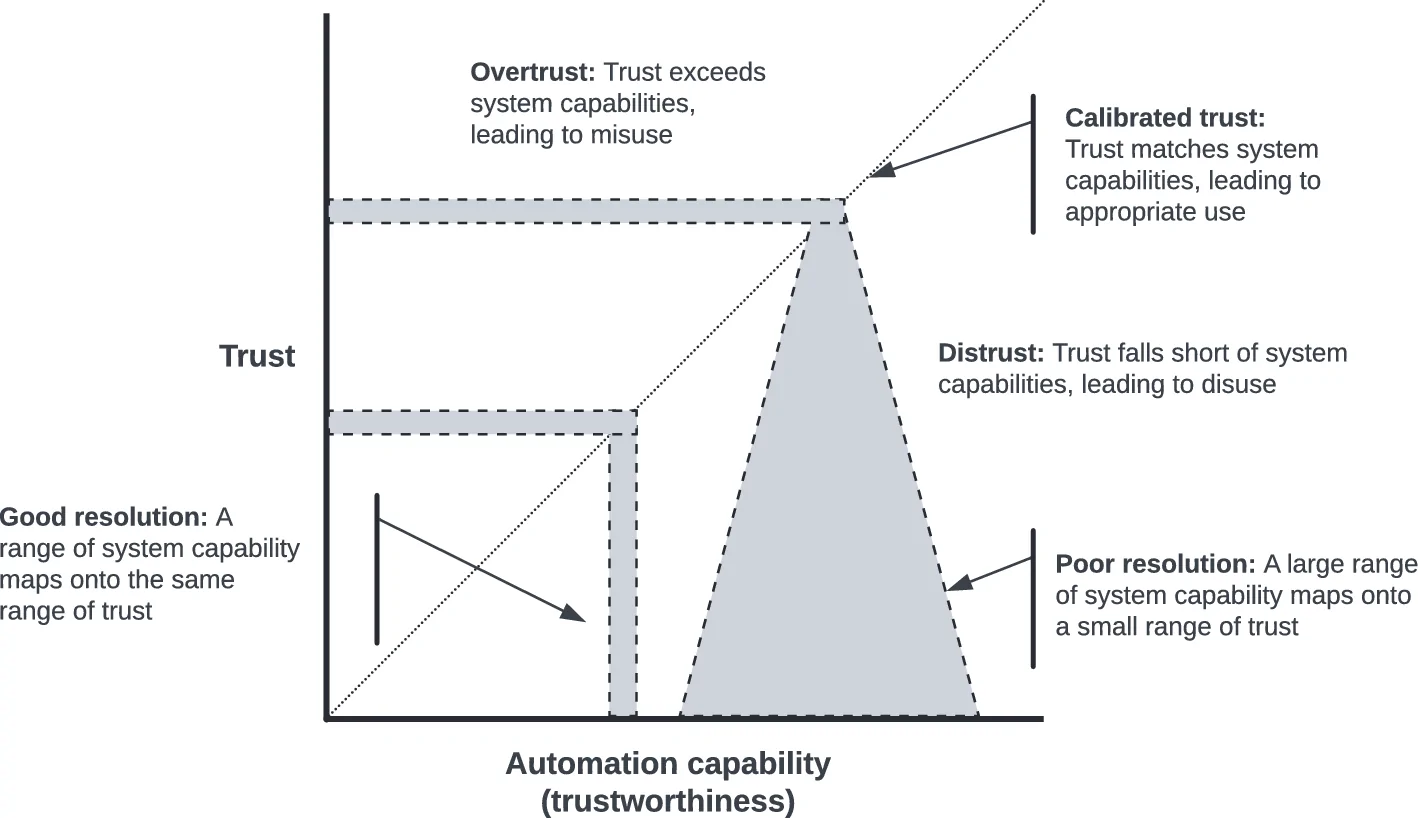
Appropriate Reliance. Figure re-published with permission from Lee and See (2004)

In cases where the system informs about the ultimate decision (such as whether to engage an enemy vehicle), the effectiveness of explanations can be measured objectively by *the switch percentage paradigm* (Ribeiro et al., 2016), which has the goal of determining the enabling factors for optimizing appropriate reliance (Lee & See, 2004). Appropriate reliance (also known as *trust calibration*) is represented by the degree to which the deployer follows the system’s recommendation when it is accurate, combined with the degree to which the decision maker deviates from it when it is inaccurate. Thus, appropriate reliance is akin to a delicate tight-rope walk that balances between overtrusting and distrusting the system (see Figure 7). A higher switch percentage indicates more trust in the system, which when coupled with a more effective or accurate system will lead to enhanced decision making. In military applications, explainability plays a crucial role in this calibration process, as commanders need to trust the system’s recommendations while understanding the reasoning behind them, especially in high-stakes contexts. Inversely, a lower switch percentage indicates lower the trust in the system, which will lead to enhanced decision making only if the system is less effective or accurate than the decision maker independently.

Consequently, the switch percentage offers insights into how to optimize appropriate reliance, namely by designing the system to incorporate the enabling factors that increase the switch percentage, while, of course, maintaining the accuracy of the system. In high-stakes environments like military decision making, explainability is essential for narrowing the responsibility gap, as unclear or opaque recommended actions from decision policies learned via RL can hinder efforts to assign accountability, especially when outcomes are ethically significant or when failures occur. One critical limitation to this paradigm, especially in the context of military applications, is that it does not take into account how risk and the associated stakes modulate trust-based behaviors. That said, it does not make any prior assumptions about the importance of risk, so that the paradigm is theoretically neutral in this regard. So far, this has only empirically been tested for supervised learning, which highlights a lack of thorough empirical studies in XRL.

Approaches to XRL can be broadly classified along two axes: *scope* and *time*. They can generate *local* explanations centering on a single action or *global* explanations justifying the general behavior. The time dimension focuses on how and when the explanation is generated relative to the RL agent’s training. *Intrinsic* methods use the same mechanism as the RL agent are thus generated simultaneously, while *post-hoc* explanations are generated by a mechanism added to the RL agent. For a more detailed taxonomy see for example Milani et al. (2023b).

A review of the empirical research employing the switch percentage paradigm in supervised learning reveals three important enablers of appropriate reliance. These enablers are summarized by the Reasons, Counterfactuals and Machine Confidence (RCC) approach, articulated by the theory of human-AI Epistemic Quasi-Partnerships (EQP) (Dorsch & Moll, 2024). The idea of reasons and counterfactuals can be found in the XRL literature as well, providing factual and counterfactual explanations for actions (Milani et al., 2023a).

Vouros (2022) offers an overview of the state of the art and challenges in XRL, with specific focus on the needs of deployers. The challenge in making learned decision policies explainable primarily arises from the complexity, which encode information in ways that are difficult for decision makers to understand. However, a more explainable RL agent could support adaptability by helping operators interpret decisions in fast-changing environments, which is essential in military contexts. The author argues that the potential to meet this challenge lies in developing interpretable decision policies and employing methods such as distillation and mimicking to approximate complex models, incorporating model confidence measures, providing local explanations, and offering counterfactual information to enhance the explainability of RL agents.

In the context of military applications, XAI in DSSs is crucial for ensuring that commanders can understand and rely on the system’s recommendations. De Reus and colleagues (De Reus et al., 2021, 2024; Maaiveld et al., 2024) exemplify this by developing an AI-based tool for generating military COAs and explaining them in a decision-maker friendly way. The exact application involves genetic algorithms to optimize COAs based on simulated effects and providing explanations anchored to geospatial elements and tactical features. This approach supports not only transparent decision making but also human-machine teaming, allowing commanders to integrate the RL agent’s insights into broader operational strategies. Thus, this model-agnostic approach ensures decisions are transparent to deployers by enhancing appropriate reliance and usability of DSSs in military decision-making processes. Further, explainability helps ensure compliance with laws of war and rules of engagement, demonstrating that decision policies align with legal and ethical constraints in military contexts.

## Review methodology

In this section, we present our methodology, based on the Preferred Reporting Items for Systematic Reviews and Meta-Analyses (PRISMA) guidelines (Page et al., 2021). First, in Subsection 3.1 we discuss how articles were identified using various databases and the limitations of our approach. Next, in Subsection 3.2 we discuss how this initial set was screened to contain only relevant articles, and in Subsection 3.3 how the final set of eligible articles was generated. Finally, each article in the final set was classified using a set of 19 characteristics, which are introduced in Subsection 3.4.

### Identifying articles

To identify relevant articles, the search pattern used in Rempel and Cai (2021) was used as a starting point. In addition, three core topics relevant to this article’s aim were identified, namely (1) *decision support*, (2) *reinforcement learning frameworks to solve sequential decision problems*, and (3) *defense and security*. Given the aforementioned search pattern and these topics, the following search pattern was constructed:

(“decision support” OR “decision support technology” OR

“decision support system” OR “behavior automation” OR

“behavior modeling”)

AND

(“reinforcement learning” OR “approximate dynamic

programming” OR “adaptive dynamic programming” OR

“neuro-dynamic programming” OR “sequential decision

making”)

AND

(military OR army OR navy OR “air force” OR “defense force” OR “armed services” OR security OR “national

safety” OR maritime OR disaster OR crisis OR “civil

defense”)

In addition, only research published from the year 2000 to when the search was conducted, published through conferences or journals, or unclassified works found in national databases was considered within scope. Lastly, at least one of the nations that participated and actively contributed to the SAS-181 RTG had to be represented in the authorship of the found article. The combination of the search pattern and these bounds were used to search within titles, authors, abstracts, and keywords for an initial set of articles.

Given the breadth of nations participating in the SAS-181 RTG, each nation individually conducted a search. The set of databases, citation databases, and search engines used by each nation is listed in Table 4. As each database and search engine has its own interface and restrictions on how a search may be performed, the above query was modified as necessary given interface restrictions. For example, Canada conducted a search using its national database, the Canadian Defence Information Database, which has a 100 character limit on the search pattern. The result of this limitation was that multiple searches were conducted and manually combined. As 10 different sources were used in total, the queries used for each are not listed here for the sake of brevity.

**Table 4:** List of national databases (ND), citation databases (CD), and search engines (SE) used each by nation to identify articles. The month and year that searched were conducted are listed. Nations are listed in alphabetical order

Nation	Database / Search engine	Type	Search date
Canada	Canadian Defence Information $\hbox {Database}^{1}$	ND	May 2023
	Web of Science	CD	June 2023
Germany	$\hbox {MErk}^{2}$	ND	February 2024
	Google Scholar	SE	February 2024
Finland	Doria $\hbox {(National Library of Finland)}^{3}$	ND	June 2023
	Google Scholar	SE	June 2023
France	Manual search performed by representatives, no access to national databases	-	June 2023
Netherlands	TNO digital $\hbox {library}^{4}$	ND	April 2023
	Scopus	CD	April 2023
	Web of Science	CD	April 2023
	Google Scholar	SE	April 2023
Norway	FFI $\hbox {database}^{5}$	ND	June 2023
	Web of Science	CD	June 2023
	Google Scholar	SE	June 2023
Sweden	rapportsö$\hbox {k}^{6}$	ND	October 2023
	Manual search performed by representative	-	October 2023
United Kingdom	$\hbox {Athena}^{7}$	ND	June 2023

^1^https://candid.drdc-rddc.gc.ca/cowdocs/cow1_e.html. Publicly available, access restricted.

^2^ Not publicly available.

^3^ doria.fi. Publicly available.

^4^ repository.tno.nl. Publicly available.

^5^ www.ffi.no/en/publications. Publicly available, access restricted.

^6^https://www.foi.se/en/foi/reports.html. Publicly available.

^7^ Not publicly available

It is important to note three limitations of how these searches were conducted and how these limitations bias the results and impact reproducibility. First, searches were limited to those articles in which at least one author was from a nation that participated and actively contributed to the SAS-181 RTG. Although this limitation was specified in the group’s objectives (see Section 1), it does restrict the set of articles included in the review. Thus, this review neither represents the complete state of RL for decision support across NATO nor worldwide. However, it does represent a sample of NATO member states that covers the spectrum in terms of defense expenditure, equipment expenditure, and includes NATO’s two newest members, Finland and Sweden (NATO, 2024a).

Second, while several databases are publicly accessible, they require an account that is restricted to specific groups of individuals—or in the case of the Athena database, can only be accessed via government computer networks. For example, in order to access the Canadian Defence Information Database, an individual must either be a member of the Canadian Armed Forces, a staff member of the Canadian Department of National Defence, or a current or potential defense contractor. While the advantage of using national databases is that it provides a set of articles otherwise not accessible, the disadvantage is that this review’s results may not be reproducible without assistance from specific nations. Last, the third limitation is that searches conducted using national databases were limited to those articles that are unclassified and publicly releasable. Research may exist at higher classification, and thus the complete set of relevant articles from each nation may be much larger.

### Screening

Following this initial article collection, each nation’s set of articles were filtered to determine those that focus on: (1) providing support to decision makers; (2) use RL; (3) are within the defense and security context, and (4) provide sufficient information on methodology. This was done by having each nation’s participants in the SAS-181 RTG review each article in their nation’s initial article collection and remove those that did not focus on the aforementioned criteria. The remainder of this section describes each of these four screening criteria and provides examples of articles that were screened out and why.

**Decision support**: The majority of research that applies RL in the defense and security domain does not use it for decision support, but rather for automation of behavior. It may also be that the authors mention the possible utilization of their results for DSSs, but the goal of their research was not specifically aimed at DSSs. For such cases, we judged whether the research is sufficiently close for being usable in a DSS and thus within scope of this review.

One such example that was filtered out is the work done by the Netherlands, Norway, Canada, and Sweden (Roessingh et al., 2017), where RL is used to learn a decision policy to perform the bounding overwatch task. This is used for automating the behavior of the simulated entities rather than supporting a decision. The military personnel will not consult a DSS to get support on how far to run and when to halt. One may of course argue that such behavior will make military simulations more realistic, which on a larger scale are used for decision support. As in this example decision support was not mentioned, we chose to not include it in this review. Other examples of articles removed for not meeting this criterion include those learning autonomous dogfighting behavior for aircraft (Saldiran et al., 2024; Toubman, 2020), the development of an autonomous cyber threat agent (Karlsson & Holm, 2024), and the control of a camera for better target recognition (Näsström et al., 2022).

**Reinforcement learning**: Adhering to the second selection criterion, some research on military DSSs are excluded because they do not apply RL, but instead other techniques, such as genetic algorithms or supervised learning, that use simulations as their basis as well. Although genetic algorithms gradually learn behavior using a simulator and a reward signal, they do not explicitly try to find the correlation between observed state and the reward. Although interesting, those works fall outside the scope of this review.

An example of an article that was removed from the set reported by the Netherlands is Schadd et al. (2022b), which aims at eliciting the reward function from the commander of a mission from natural language. In this case, a commander’s intent is translated into a computable function and the system subsequently uses a genetic algorithm to find COAs to maximize this function. Also removed was research by Schadd and de Reus (2023). Similarly, this research uses a genetic algorithm to plan obstacle effects in a defensive mission such that the effects maximize the delay of the adversary, making use of advantageous terrain features.

**Defense and security**: The research should be situated firmly within the defense and security context to be relevant. Although the domain is quite broad, research should specifically address one or more defense and security challenges. For example, some articles in the initial set concerned games related to defense and security—such as work from the Netherlands that focused on defending a space fortress (Van Oijen et al., 2019) and research by Sweden on playing Risk (Limér et al., 2020)—however these are primarily intended for entertainment purposes rather than military relevant. Thus, such articles were excluded from further consideration.

**Methods**: Some articles are excluded because essential parts of the method or results are not disclosed. For example, an article within the initial set provided by Sweden (Brynielsson et al., 2022) discussed patrol path planning, but many details were not provided and thus it was deemed not eligible to be included in the final set.

### Generating the final set

Following the filtering of articles as discussed in the previous section, two further steps were taken to generate the final set of articles. First, incoming and outgoing references of the filtered set of each nation were scanned for additional relevant research. Second, each nation added articles that were collected using the networks of individuals participating in the SAS-181 RTG. These additional articles were screened so that they adhered to the same criteria as discussed in Subsection 3.1 and Subsection 3.2. Lastly, relevant articles authored by members of the SAS-181 RTG that were in review when the search queries were executed, and published prior to this article’s submission in February 2025, were added to each nation’s set. The results of all participating nations were combined to create a final set of relevant articles.

### Classification of the literature

To gain an overview of the research field and classify the articles, it is necessary to abstract details of each article and make aspects comparable. To do so, we chose characteristics with the aim to understand what is the focus of the existing literature, and gaps that exist. The chosen characteristics are both specific to our domain of study and demarcate the discovered works.

As part of the initial compilation of relevant characteristics, we scanned existing reviews on RL for DSSs, both in and outside of the military domain. Gautron et al. (2022), who reviewed crop management, considered characteristics such as *Decision Type*, *Number of Objectives*, *Learning Algorithm(s)*, *Environment Dynamics* and *Uncertainties Regarded*. Set within a military context, Rempel and Cai (2021) explicitly include a *Policy Category* characteristic, and discussed aspects such as the *Application Domain* and the *Domain of Warfare*. Heilala et al. (2010), who reviewed manufacturing operations planning, included a characteristic focused on the *Human Decision-Making Time Frame*. In addition, *Evaluation* and *Maturity* are characteristics that are frequently discussed in literature (Eckardt et al., 2021; Gautron et al., 2022; Merkert et al., 2015).

This set of characteristics were modified and extended throughout discussions between the authors. *Country of Origin* was identified as a relevant characteristic as this review builds on national reviews. From a decision support perspective, *Simulation Horizon* and *Type of Simulator* are key characteristics as they relate to aspects such as planning, available information, model resolution, prediction accuracy and interoperability. Additionally, we chose to distinguish between direct and indirect *Policy Usage* and capture whether an *Algorithmic Explanation Capability* was employed. Finally, this set was complemented with characteristics describing *Uncertainty Modelling* and *Multi-Agent Reinforcement Learning* to reflect these aspects of RL.

We regard that this final set of 19 characteristics provides appropriate depth for creating a broad picture. Below we provide a rationale for choosing each characteristic. In the brackets we enumerate all eligible values for each characteristic, where the set of eligible values where chosen to generally match the content of the articles. For some characteristics, *None* is a legal value and it indicates that none of the eligible values match the article.

In addition, we mapped the characteristics to our extension of Powell’s unified framework for sequential decisions as depicted in Figure 6. To do so, for each characteristic two questions were posed: (1) Is the characteristic more strongly associated with the *model first* steps or the *then solve* steps?, i.e., the left three or the right three hexagons in Figure 6; and (2) To which of the chosen three steps does the characteristic best fit?^16^ Note that *Country of Origin* was not mapped to a step as it is neither associated with modeling nor solving a given decision problem. The mapping is indicated by the preceding hexagon in the list below, for which the depicted letter corresponds to the first letter of a step in Figure 6.

- ∙ **Country of Origin** [] This characteristic enables us to label the origin of the research.
**Decision Support Topic** [Based on found literature] This is the decision that is to be supported. Examples are *IED awareness* or *disaster management*. This characteristic was chosen to be able to show the broad spectrum of possible applications.
**Simulation Horizon** [Seconds (S) / Minutes (M) / Hours (H) / Days (D) / Months (Mo) / Years (Y)] This indicates the time frame that the simulations cover. For example missile defense may be minutes, while procurement might be years. With this characteristic we get an overview of how far ahead current simulations dare to extrapolate.
**Human Decision-Making Time Frame** [Seconds (S) / Minutes (M) / Hours (H) / Days (D) / Months (Mo) / Years (Y)] An indication of how long a human has available to make the decision. Having more or less time is relevant, as it restricts the possibilities of understanding and trusting the RL-based DSS for acute decisions.
**Application Domain** [Defence Planning (DP) / Force Generation (FG) / Force Projection (FP) / Force Employment (FE)] Defense planning concerns what capabilities the military force should possess in the future and is part of the NATO terminology (NATO, 2024b). For the terms, generation, projection and employment, we follow the definition of the Department of the Army (2019b).^17^ It is of interest whether RL-based DSSs cover all application domains.
**Decision Type** [Strategic (St) / Operational (O) / Tactical (T)] The military distinguishes three levels of decisions that each come with their own challenges. By using this characteristic, it becomes clear whether each decision level is addressed with RL-based decision support.
**Domain of Warfare** [ /  /  /  /  / Multi-Domain Operations (MDO)] The military is historically divided into the *land*, *air* and *maritime* domains, and have only recently expanded with *space* and *cyberspace* (NATO, 2023). Missions that are orchestrated over multiple domains, synchronized with non-military activities, are indicated with the term *MDO*.^18^ In cases of articles that are generic in nature, such as procurement of equipment for which the mission environment is not known yet, we chose to label these as MDO. As each domain has their own challenges, e.g., terrain is less relevant for air operations, this characteristic was chosen to gain insight into the utilization of RL-based decision support in these domains.
**Policy Usage** [Direct (Di) / Indirect (I)] As discussed in Subsection 2.2, the policy itself may be used to support the decision (direct), or it may be used within a larger decision support framework (indirect). With this characteristic, the most prominent use of the learned policies is identified.
**Multi-Agent Reinforcement Learning (MARL)** [Single Policy (SP) / Collaborative Policies (CP) / Adversarial Policies (AP)] For the learning it makes a large difference whether there is one agent, or whether collaboration or competition between multiple agents is occurring. We consider the system as a multi-agent system only when multiple agents are *learning* at the same time, and each is learning its own policy. This is in line with the definition of MARL (Buşoniu et al., 2010; Canese et al., 2021; Lowe et al., 2017; Zhang et al., 2021). The agents may be collaborating, be in competition, or somewhere in between.
**Environment Dynamics** [Static (Sta) / Dynamic (Dy)] This indicates whether the traits of the uncertainties or the dynamics change over time. For example, while the laws of physics do not change for space operations, an earthquake or heavy storm may change the terrain significantly. As machine learning and RL are traditionally applied in static environments due to controllability and predictability, it is relevant to investigate whether RL is applied in dynamic environments in the military domain.
**Number of Objectives** [Single Objective (SO) / Multi Objective (MO)] It is much harder to obtain an optimal, or near-optimal, decision policy when multiple objectives are considered. An example of a multi-objective task in the military is to defend an important bridge while reducing the amount of personnel losses. Typically multiple objectives are translated to a single objective using a weighted sum of the objectives. We regard this case as single objective. It is multi-objective when multiple feedback signals are used, in essence, one for each objective. MORL is the learning of policies that optimize multiple feedback signals simultaneously (Moffaert & Nowé, 2014).
**Uncertainties Regarded** [Observational Errors (OE) / Exogenous Uncertainty (EU) / Prognostic Uncertainty (PU) / Inferential Uncertainty (IU) / Experimental Variability (EV) / Model Uncertainty (MU) / Transitional Uncertainty (TU) / Control Uncertainty (CU) / Communication Errors (CE) / Algorithmic Instability (AIn) / Goal Uncertainty (GU) / Political/Regulatory Uncertainty (PRU) / none (-)] There exist many types of uncertainties in the real world, and each of them may be addressed in some form in a DSS. Powell (2022) identifies twelve such uncertainties, as listed in Table 1. This characteristic indicates whether the DSSs takes into account uncertainties.
**Uncertainty Modelling** [Historical Data (HD)/ Subject Matter Expert (SME) models / Monte Carlo (MC) / none (-)] As uncertainty often plays a large part in the problem that is being modeled, and because it has a large influence on the ability to learn a policy, this characteristic has been included. Typically uncertainties are modeled using randomized events because otherwise the RL agent will learn that the same event always will happen. The event probabilities may be based on historical data, on judgments of SMEs, or simply chosen by the designers (Monte Carlo). This characteristic has a relation with the Uncertainties Regarded characteristic. For example, not knowing an adversary’s location, while being sure that there is an adversary, is a type of exogenous information (see Table 1). This uncertainty may be modeled by randomly placing the adversary on the map (Monte Carlo), until the exact position of the adversary is known.
**Policy Category** [Direct Lookahead Approximation (DLA) / Policy Function Approximation (PFA) / Value Function Approximation (VFA) / Cost Function Approximation (CFA) / Hybrid (HYB)] This characteristic describes how the policy makes a decision for a given state. Powell (2022, Ch. 11) classifies any decision policy into four distinct groups, and any hybrid variant. See Table 2.
**Learning Algorithm(s)** [Q-learning (QL) / Approximate Value Iteration (AVI) / Proximal Policy Optimization (PPO) / State-action-reward-state-action (SARSA) / Best Response with Thompson Sampling (BR-TS) / Monte Carlo Tree Search (MCTS) / Monte-Carlo Reinforcement Learning (MCRL)] This characteristic provides a high-level overview of how the RL agent learns well-performing behaviors. This characteristic is correlated to the Policy Category characteristic, but the main difference is that the Policy Category characteristic defines the class of policy that is learned, while the Learning Algorithm(s) characteristic describes *how* it is learned. Taxonomies and classification of RL algorithms are proposed in several articles.^19^ We chose to use categories of common terms encountered in RL literature.^20^ It should be noted that for a given algorithm multiple variants exist in the literature beyond those listed. To enable comparisons between articles, we represent all variants of a type of algorithm with their common label, i.e., all variants of PPO, including self-play (Berner et al., 2019), are categorized as PPO. When an article compares multiple learning algorithms, only the best-performing one is reported. However, if an article applies two different learning algorithms to two distinct sequential decision problems, then both are reported. In some cases the label used does not indicate a learning algorithms in itself, but rather a framework in which learning can be take place. For example, MCTS generates data that is used for training a neural network to help in different forms during the playouts (Silver et al., 2018).
**Type of Simulator** [Open Source (OS) / Bespoke (B) / Commercial off the Shelf (COTS) / Government off the Shelf (GOTS)] The simulator is the backbone of an RL-based DSS. The complexity of missions in the military is typically so high, that it is nearly impossible to include all aspects. If comprehensive simulations are attempted, the simulator may become too slow to be useful for RL purposes. Furthermore, the design of the simulator and its interface affects the available options for the learning algorithm. It is therefore of interest to investigate what kind of simulators are used within RL-based DSSs. We chose frequently used software categories as possible values.^21^
**Evaluation** [Hypothetical (Hy) / Qualitative (Qual) / Quantitative (Quant) / none (-)] Validating military DSSs is not a straightforward task, as typically not much data is available and no standardized TEVV methodology for RL-based DSSs exists. Therefore it is of interest what type of TEVV activities are performed, if any (see Subsubsection 2.3.5). Quite often learning curves are shown, or the approach is compared to other approaches, such as a hard-coded default policy. We call these cases a hypothetical validation. A hypothetical validation however does not say anything about whether the resulting policy is “good enough” from the user’s perspective for the required decision support. This can be validated either qualitatively by judgements from domain experts, or quantitatively by using a defined benchmark or measuring the policy’s performance in the real world.
**Maturity** [Theoretical (Th) / Proof of Concept (PoC) / Field Tested (FT) / Operational (O)] While many attempts are being made to create RL-based DSSs for the military, only few actually are implemented in the operational environment. With this characteristic, an overview of the maturity of such systems becomes possible. Currently, there is no standardized TEVV methodology for RL-based DSSs, which makes it difficult to consistently assess and compare the maturity of different studies. Developing such a framework would be highly valuable, as it would provide clear criteria for evaluation, highlight gaps in assessment, and accelerate the translation of promising RL approaches into operational settings. There exist many generic methods for measuring the maturity of an approach,^22^ but as the covered research in this article can not be mapped easily to any of these scales, we opt to create the following four categories: (1) Th—theoretical work that shows the promise of RL, but does so on simplified scenarios; (2) PoC—a proof of concept that uses representative military or security scenarios and simulations, but is still tested within a lab setting. For example, when the application is using an actual military scenario in a realistic simulator; (3) FT—once the system has been tested within the field with actual users, then this level is achieved. This could for example be military exercises; (4) O—when the system is operational and being used by actual users to provide decision support without any support of the developers.^23^
**Algorithmic Explanation Capability** [ Explaining (E) / none (-) ] Methods are becoming available for explaining machine learning models, however these have yet to prove themselves in military DSSs. We therefore believe it sufficient to simply indicate whether an article made any effort at all to explain the learned policy to the decision maker. It is important to note that we do not regard an interpretation of the (general) behavior by the researcher as a valid explanation. The explanation should be performed automatically for the current specific situation that requires decision support (i.e., local explanations).^24^

In the remainder of the article, when referencing a characteristic, the name is capitalized within the sentence.

When attributing characteristics to a research article, the following procedure was followed. First, the author or national representative of the corresponding country proposed an assignment. Secondly, two representatives of different countries made an independent assessment of the characteristics for the research article. Any deviations were discussed plenary with the remaining co-authors of this article.

## Results

In this section, we present an overview of 20 application-based articles identified through the aforementioned literature search. First, in Subsection 4.1 we list and characterize each study reviewed using the set of characteristics given in Subsection 3.4. Next, in Subsection 4.2 we present a visual summary of the reviewed literature through applying dimensionality reduction, namely the Uniform Manifold Approximation and Projection technique, and qualitatively describe the resulting visualizations. Lastly, in Subsection 4.3 cross-tabulations are used to assess the relationships between articles using specific characteristics.

### Classifying existing literature

Table 5 lists the results of the manual labeling of each article using the aforementioned characteristics in the previous section. For the sake of brevity, the abbreviations as stated in Subsection 3.4 are used, and cells with a ‘-’, it indicates a ‘None’ value for that article. Articles are grouped by country.

**Table 5:** An overview of application-based articles during the period 2000-2024 that employ RL for decision support within the defense and security domain. For abbreviations, see Subsection 3.4

Reference	Country of Origin	Decision Support Question	Simulation Horizon	Human Decision-Making Time Frame	Application Domain	Decision Type	Domain of Warfare	Policy Usage	Multi-Agent Reinforcement Learning	Environment Dynamics	Number of Objectives	Uncertainties Regarded	Uncertainty Modelling	Policy Category	Learning Algorithm(s)	Type of Simulator	Evaluation	Maturity	Algorithmic Explanation Capability
^25^	NLD	Ambush Positions	H	HH	FE	T		Di	AP	Sta	SO	EU	MC	VFA	QL	B	Qual	PoC	–
^26^	NLD	Engagement Scheduling	M	S	FE	T		Di	SP	Sta	SO	EU	MC	DLA	MCTS	B	–	Th	–
^27^	NLD	IED Avoidance	H	H	FE	T		I	AP	Sta	SO	EU	-	VFA	PPO	B	–	Th	–
^28^	NLD	Concept Development	H	Mo	DP	St		I	SP	Sta	SO	EU	SME	PFA	PPO	B	–	PoC	E
^29^	CAN	Maintenance Scheduling	Y	D	FE	T		Di	SP	Sta	SO	TU	MC	VFA	MCRL	B	Quant	Th	–
^30^	CAN	UAV Routing	H	M	FE	T		Di	SP	Sta	SO	GU	-	VFA	QL	B	–	Th	–
^31^	CAN	Disaster Response	H	M	FE	T		Di	SP	Sta	SO	AIn	-	VFA	SARSA	B	–	PoC	–
^32^	CAN	Investment Planning	Y	Mo	DP	St		Di	SP	Sta	SO	EU	MC	CFA	-^33^	B	Hypo	PoC	–
^34^	CAN	Resource Assignment	Mo	H	FE	T		Di	SP	Sta	SO	EU	HD	VFA	AVI	B	Hypo	Th	–
^35^	CAN	Disaster Management	D	M	FE	T		Di	SP	Sta	SO	EU	MC	VFA	AVI	B	Hypo	Th	–
^36^	CAN	Disaster Management	D	M	FE	T	MDO	Di	CP	Sta	SO	EU	MC	VFA	AVI	B	Hypo	Th	–
^37^	DEU	COA Generation	H	H	FE	T		I	SP	Sta	SO	EU	MC	VFA	PPO	B	$\hbox {Qual}^{38}$	Th	–
^39^	DEU	COA Generation	H	H	FE	T		I	AP	Sta	SO	EU	MC	VFA	PPO	B	Hypo	Th	–
^40^	DEU	COA Generation	H	H	FE	T		I	AP	Sta	SO	EU	MC	VFA	PPO	B	–	PoC	–
^41^	DEU	COA Generation	H	H	FE	T		I	AP	Sta	SO	EU	MC	VFA	PPO	B	–	PoC	–
^42^	SWE	Decoy Allocation	S	D	FE	T		Di	AP^43^	Sta	SO	-	MC	CFA	BR-TS	B	Quant	Th	–
^44^	SWE	Air Attack Planning	H	D	FE	O		Di	AP	Sta	SO	EU	MC	DLA	MCTS	B	–	Th	–
^45^	FIN	Maintenance Scheduling	Mo	H	FG	T		Di	SP	Sta	SO	EU,TU	MC	VFA	QL, SARSA	B	Hypo	Th	–
^46^	FIN	COA Generation	H	H	FE	T		I	AP	Sta	SO	EU,AIn,OE	MC	VFA	QL, PPO	B	–	Th	–
^47^	FIN	COA Generation	H	D	FE	T		I	AP	Sta	SO	EU,AIn,OE	MC	VFA	PPO	B	–	Th	–

^25^ De Reus et al. (2021)

^26^ Koole (2021)

^27^ De Heer et al. (2019)

^28^ Reinten et al. (2024)

^29^ Barde et al. (2019)

^30^ Bélanger et al. (2008)

^31^ Castellanos et al. (2018)

^32^ Fisher et al. (2015)

^33^No real learning takes place, as all eligible options are simply enumerated.

^34^ Powell et al. (2011); It should be noted that Boukhtouta et al. (2010) is an internal Defence Research and Development Canada (DRDC) report that was published prior to Powell et al. (2011). As these references both cover the same information, we chose to only include Powell et al. (2011) in the table as it is publicly available.

^35^ Rempel et al. (2021)

^36^ Rempel and Shiell (2023)

^37^ Doll et al. (2021)

^38^Not indicated in the article. The value is based on personal communication with author

^39^ Möbius et al. (2022)

^40^ Möbius et al. (2023a)

^41^ Möbius et al. (2023b)

^42^ Brynielsson et al. (2023)

^43^We classify this as adversarial, because a human is playing the adversary during the learning.

^44^ Pelz and Bengtsson (2023)

^45^ Mattila and Virtanen (2011)

^46^ Vasankari and Saastamoinen (2024)

^47^ Vasankari and Virtanen (2025)

Given the summary of the articles in Table 5, five insights are evident. First, the majority of articles focus on the Force Employment application domain and on decisions at the tactical level. Second, all articles work with static Environment Dynamics and are modeled as single objective problems (or are multi-objective problems, but are translated into a single-objective by means of a weighted sum). Third, VFA is the most often used Policy Category. Fourth, developing a bespoke simulation is the standard approach towards implementing the transition function and arrival of exogenous information. Lastly, the overwhelming majority of articles do not utilize an algorithm to explain the learned policy to a decision-maker (however it should be noted that manual interpretation may be done, such as in Rempel et al. (2021)). Such trends and gaps are further discussed in Subsection 5.1.

### The research landscape

As an effort to gain an overview of the articles listed in Table 5, the Uniform Manifold Approximation and Projection (UMAP) algorithm—a general purpose, computationally efficient, non-linear dimensionality reduction technique that seeks to preserve global structure of high-dimensional data (McInnes, 2018; McInnes et al., 2020)—was used to find a low-dimensional representation of the articles and the relationships between them. In general, UMAP works by first finding a fuzzy topological representation of the data, and then optimizing a low-dimensional representation such that it is a close match to the high-dimensional representation as measured by cross-entropy. Though it is a relatively new method, UMAP has been used in a variety of domains, including bioinformatics, materials science, and machine learning (see McInnes et al. (2020, p. 2) for references). While these applications demonstrate UMAP’s applicability to large data sets, research by Lin et al. (2018) demonstrates its usefulness for smaller data sets, such as in this review.

In order to use UMAP, the data set in Table 5 was separated into two subsets: the first being the articles as described by nominal characteristics; and second the articles as described by ordinal characteristics. For the latter, the set of ordinal characteristics included *Simulation Horizon*, *Human Decision-Making Time Frame*, *Decision Type*, *Maturity*, and *Evaluation*. The remaining characteristics were identified as nominal, with the exception of *Country of Origin* and *Decision Support Topic* which were excluded as both do not impact how a sequential decision problem is either modeled or solved.

Next, the nominal data set was pre-processed using one-hot encoding to prepare it for use by UMAP. Likewise, the ordinal data set was transformed using ordinal encoding. For articles that included more than one source of uncertainty, the list of uncertainties was coded as a combination. However, for those articles that employed more than one learning algorithm to seek a decision policy, namely Mattila and Virtanen (2011) and Vasankari and Saastamoinen (2024), a separate encoding for each described algorithm was created.

UMAP was used with the dice metric to measure distance between articles in the nominal data set, the Manhattan metric for the ordinal data set. The union of the two resulting UMAP models was then generated as described by McInnes (2018). The result is a two-dimensional projection in an Euclidean space, where each point represents an article (with the exception of Mattila and Virtanen (2011) and Vasankari and Saastamoinen (2024)). Articles are then labelled based on any of the 19 characteristics described in Subsection 3.4.

**Fig. 8:**
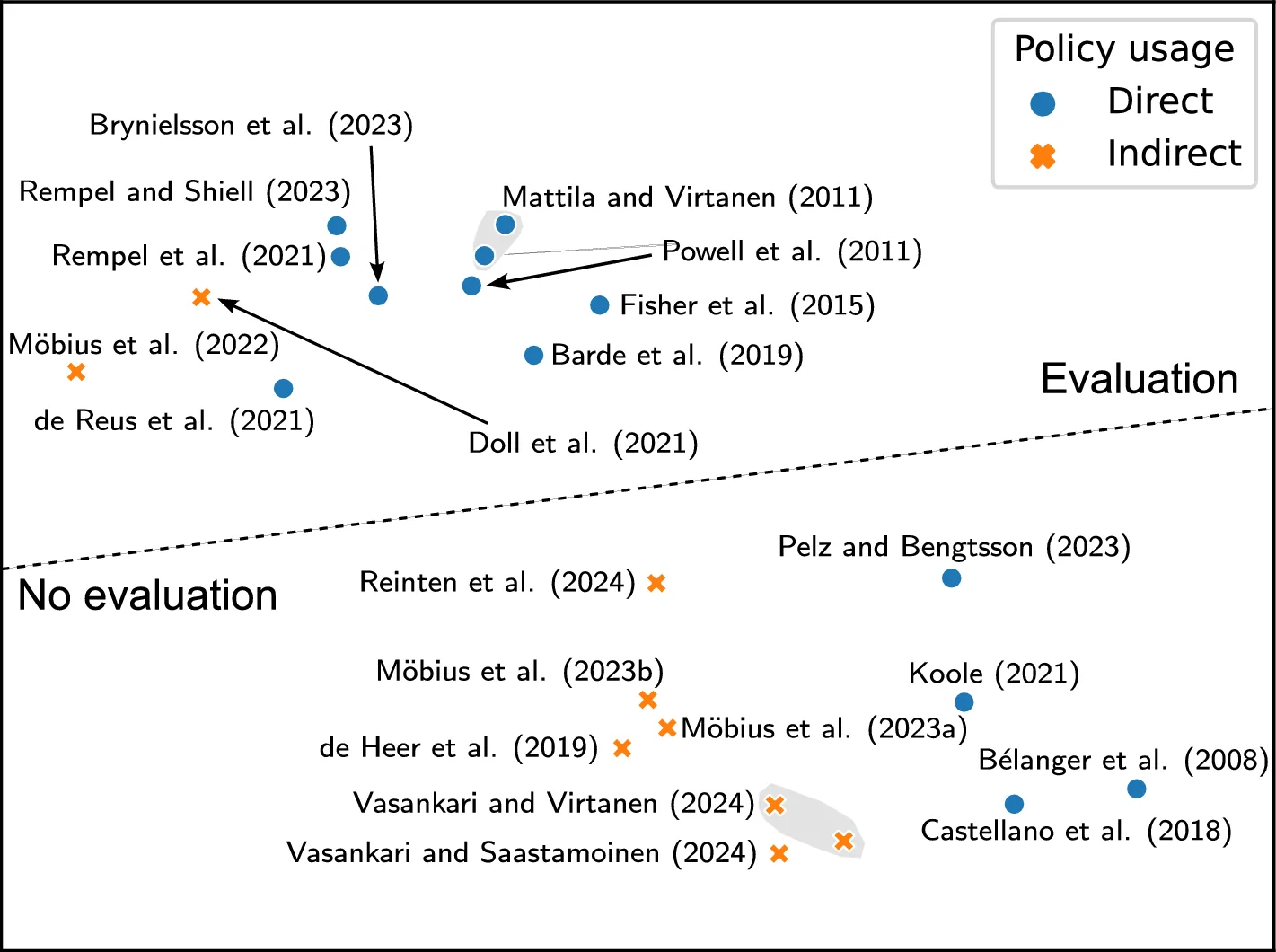
UMAP representation of the 20 articles listed in Table 5. The gray regions represent those articles— Mattila and Virtanen (2011) and Vasankari and Virtanen (2025)—that studied their respective problems using more than one RL algorithm without seeking the one that performs the best

The results of applying UMAP are depicted in Figure 8. We found that the 20 articles were separated into two clusters, with the main distinction being whether or not evaluation was conducted. The lower cluster (below the dashed line) is those articles that did not perform evaluation, while those in the upper cluster (above the dashed line) performed some type of evaluation. The structure within each cluster is further explained by the *Policy Usage* characteristic. This characteristic separates each cluster into two sub-clusters, one on the left in which *Policy Usage* is indirect and one on the right in which it is direct. While not shown here, we also found that the *Application Domain*, *Domain of Warfare*, *Simulation Horizon*, and *Uncertainties Regarded* characteristics provided insight into the structure within each cluster, although at a more granular level than depicted in Figure 8. Specifically, while both clusters are heterogeneous in terms of these four characteristics, articles that are similar tend to be grouped together.

### Specific comparisons

In the previous section, a global landscape of the found research articles was presented. In this section we focus on specific comparisons between selected characteristics in order to highlight insights that are not apparent in Figure 8. A total of four comparisons are presented.

An overview of how mature various decision support applications are for different domains of warfare is presented in Table 6. It can be seen that the majority of research published to date is focused on the land domain, followed by the air and navy domain. None of the RL-based DSSs described in the articles are considered to be field tested or operational.

**Table 6:** The Maturity of research efforts for each Domain of Warfare. Abbreviations are Theoretical (Th), Proof of Concept (PoC), Field Tested (FT) and Operational (O)

	Maturity					
		Th	PoC	FT	O	**Total**
Domain of Warfare		4	5			9
		5				5
		3	1			4
		1				1
	**MDO**	1				1
						0
	**Total**	14	6	0	0	20

Table 7 lists what uncertainties where regarded in each domain of warfare. We observe that exogenous uncertainty is almost always (16 out of 20) taken into account. A frequently seen uncertainty from the force employment realm are the unknown actions of the adversary, such as the position of units (fog of war). Other uncertainties are only taken into account occasionally. It should be stressed that there are not sufficient data points to draw conclusions about what type of uncertainties are more prevalent in a certain domain.

**Table 7:** The number of articles that regard a form of uncertainty in each Domain of Warfare. Abbreviations are Exogenous Uncertainty (EU), Transitional Uncertainty (TU), Goal Uncertainty (GU), Algorithmic Instability (AIn) and Observational Errors (OE). Please note that a single article can cover uncertainties of different forms and count for multiple columns. Forms of uncertainty that are never addressed are omitted

		Articles that regard					
		EU	TU	GU	AIn	OE	**# articles**
Domain of Warfare		7	1		1		9
		4	1	1			5
		4			2	2	4
							1
	**MDO**	1					1
	**Total**	16	2	1	3	2	20

Table 8 compares the Simulation Horizon to the Human Decision-Making Time Frame. A significant number of articles (7 out of 20) considered decisions that have to be taken within an hour of time, and which are supported by simulations that also span hours. A positive correlation between these two characteristics is expected as the simulation horizon should match the decision-making level, as described in Subsection 2.1. When there is much time available for making a decision, then typically it concerns a decision that applies an effect over a long time as well. When short simulations are used, then also typically a quick decision has to be made. Table 8 shows this correlation to some extent, with three larger deviations. On the one side, with a significantly longer Human Decision-Making Time Frame compared to Simulation Horizon, we see applications in concept development (Reinten et al., 2024) and defense against cyber attacks (Brynielsson et al., 2023) standing out. On the other side, with a significantly longer Simulation Horizon compared to Human Decision-Making Time Frame, we see an application in maintenance scheduling (Barde et al., 2019). For these three specific examples that do not follow the general correlation, the choices of the Simulation Horizon and Human Decision-Making Time Frame are justified. For example, cyber attacks happen in (milli-)seconds, and choosing a robust defense happens before the actual attack.

**Table 8:** An overview of how simulation time compares to the human decision-making time frame. Abbreviations are Seconds (S), Minutes (M), Hours (H), Days (D), Months (Mo) and Years (Y)

		Human Decision-Making Time Frame						
		S	M	H	D	Mo	Y	**Total**
Simulation Horizon	S				1			1
	M	1						1
	H		2	7	2	1		12
	D		2					2
	Mo			2				2
	Y			1		1		2
	**Total**	1	4	10	3	2	0	20

Table 9 seeks to identify in what situations the policies are being used directly or indirectly. We see a slight preference to a direct use of the learned policy (12 vs 8). It is furthermore noticeable that when using the policy directly, usually it concerns single-agent learning. In comparison, when the policy is used indirectly, then typically an adversarial learning approach has been applied.

**Table 9:** An overview of how policies are learned in terms of multi-agent learning for difference variants of indirect and direct policy usages. Abbreviations are Multi-Agent Reinforcement Learning (MARL), Single Policy (SP), Collaborative Policies (CP), Adversarial Policies (AP), Direct (Di) and Indirect (I)

Policy Usage		MARL			
		SP	CP	AP	**Total**
	Di	8	1	3	12
	I	2		6	8
	**Total**	10	1	9	20

## Discussion

Based on the review presented in Section 4, this section begins with a discussion of the trends and gaps in the current research in Subsection 5.1. Following this, Subsection 5.2 describes four challenges with regards to employing RL for decision support in a defense and security context—the complexity of scenarios and operations; the availability of data; connecting with simulators; and connecting with decision makers. For each challenge, we provide a recommendation how it may be addressed.

### Trends and gaps

As listed in Table 5, force employment is the most researched Application Domain with 17 out of 20 articles focusing on this area. The remaining three articles are split between defense planning, specifically battlefield information concepts (Reinten et al., 2024) and investment planning (Fisher et al., 2015), and force generation where Mattila and Virtanen (2011) explore maintenance scheduling of fighter aircraft to ensure sufficient availability. This segmentation aligns with that reported by Rempel and Cai (2021), who reviewed ADP applications within military operations research and found that 15 of 18 articles reviewed focused on force employment, with the remaining three categorized as defense planning and force generation. It is worth noting that neither this review nor that by Rempel and Cai (2021) found any articles that discuss force projection despite the potential of applying RL for developing near-optimal policies for moving forces.

When comparing to Rempel and Cai (2021), the topics studied within the force employment domain differ. In this review, articles addressed decision problems related to Improvised Explosive Device (IED) avoidance, disaster response / management, and COA generation, whereas in Rempel and Cai (2021) the majority were related to combat medical evacuation, missile defense, and airlift. While there is no singular reason for the predominance of force employment across the two collections of articles, it is worth noting that many decision support applications in force employment rely on simulators, and thus these applications are well positioned to leverage RL. Additionally, modeling of force employment decision problems, such as engagement scheduling and COA generation, benefit from the large body of research on RL for computer games due to their similarity.

When considering the Domain of Warfare characteristic, only one article studied an MDO, specifically a major maritime disaster scenario in which both maritime and air assets are tasked to respond (Rempel & Shiell, 2023). A reason for the minimal presence of MDO applications may be the complexity involved (Baker et al., 2020), both in terms of the required simulations and uncertainties to be modeled, i.e., the transition function and exogenous information, as compared to single domain operations. From an RL perspective, as the operation becomes more complex so does the sample and computational complexity, making it more difficult to learn a near-optimal policy (Kakade, 2003).

The Policy Category characteristic is dominated by the use of VFAs, with 15 of 20 articles employing this type of policy. However, the remaining three policy categories are utilized, with one article using a PFA (Reinten et al., 2024), two articles using CFAs (Fisher et al., 2015; Brynielsson et al., 2023), and two using DLAs (Koole, 2021; Pelz & Bengtsson, 2023). As with the Application Domain characteristic, this trend follows the one reported by Rempel and Cai (2021) who found 16 of 18 articles reviewed used a VFA policy, with the remaining using cost-function and direct lookahead approximations. Beyond defense and security, VFAs are popular in the wider RL community due to that they are well-studied, compatible with popular learning algorithms, such as Q-learning (Watkins & Dayan, 1992) and PPO (Schulman et al., 2017), and can employ various strategies to approximate the value of a future state (Powell, 2011; Sutton & Barto, 2018). This is evidenced by recent reviews, such as those in the fields of agriculture (Gautron et al., 2022) and oncology (Eckardt et al., 2021), where in the former the authors found that all articles reviewed applied variants of Q-learning, while in the latter 13 out of 16 papers based their approach on Q-learning. In addition, VFAs that employ deep neural networks have proven to be highly effective, for instance in playing games (Mnih et al., 2015), and these successes have inspired accessible tools and robust frameworks (Raffin et al., 2021; Towers et al., 2024).

A common theme across all articles listed in Table 5 is the use of bespoke simulators, and the likely reasons for this are discussed in Subsection 2.4. In short, many simulators used in a military context are built for realism and immersion rather than simulation speed, which makes them suboptimal for RL. Additionally, vendors tend not to prioritize making the necessary interfaces for exchanging data between the simulation and an RL agent. Even when the agent has access to the simulation-state data, the level of detail with which it is represented may be unusable by the policy. As discussed in Subsection 2.4, CMO is one of the commercial options that exposes an API compatible with RL. The PyCMO environment (Hua, 2023) adds another layer of convenience akin to Gymnasium, originating from a competition in applying AI for mission planning in CMO (NSIN, 2021). Another learning environment for CMO was presented by Dimitriu et al. (2024). Like in PyCMO, the authors use the structured data in CMO to create a state representation, unlike AlphaStar which takes pixel input (Vinyals et al., 2019). Importantly, this representation eliminates the need for graphical rendering, freeing up graphics processing units for tensor operations. As a proof of concept, Dimitriu et al. (2024) used PPO to learn a decision policy to coordinate a reconnaissance drone and an artillery battery, and reached proficiency after about two million time steps. It remains to be seen how well this approach scales to tactical and operational level decision making in CMO or other commercial simulators.

The ability to explain a decision policy to a decision maker is an almost unexplored feature in the reviewed literature despite the propensity of the RL community to base policies on neural networks, which are not directly interpretable. As discussed in Subsection 2.5, the choice of a complex policy may call for explanation mechanisms that then are added post-hoc. Examples of post-hoc mechanisms are featured in the interactive dashboard described in Reinten et al. (2024). In this instance, the policy is used to suggest which information concepts to use (map or compass) and the walking pace when navigating through a terrain. The dashboard enables the decision maker to experiment with the parameters of the human cognitive model and learning algorithm, retrain the policy, and visualize the preferred actions in different states along with backwards inference from actions to states. Beyond the defense and security context, Gautron et al. (2022) identify explainability capabilities as a challenge in their review of agriculture RL-based DSSs. They highlight *causal RL* as a prospective avenue for generalizing across learning problems and performing counterfactual reasoning (Dasgupta et al., 2019).

Lastly, the Maturity of RL-based DSSs in the articles reviewed was deemed to be either theoretical or at the proof of concept stage, with the majority being theoretical. Over half of the articles performed no empirical evaluation at all of the created policies and several studies only performed a hypothetical evaluation. Therefore it remains questionable whether the policies are ready to support decision makers in the field.

Nevertheless, the deployability of an RL-based DSS remains an important issue. With this in mind, the RAND Corporation recently ran a study to identify the risks involved in applying RL to decision making in complex scenarios, focusing on operational level command and control with the air tasking cycle as a use case (Bunch et al., 2024). The authors considered two options for integrating RL agents in the cycle. The most ambitious option was a single agent capable of performing all functions, which would bypass several innovation steps and accelerate development. However, a second option was recommended, which was to develop specialized agents for each step. This second option allows for technology transition to be more finely controlled and existing technologies to more easily serve as benchmarks. Additionally, given several simpler agents rather an a single complex agent may improve explainability.

### Challenges and recommendations

RL presents several fundamental challenges which are recognizable in every application domain. Examples include sample efficiency, training stability, high-dimensional state and action spaces, latency, catastrophic interference, exploration difficulties, partially observable systems, and the trade-off between exploration and exploitation (Sutton, 1992; Ding et al., 2020; Dulac-Arnold et al., 2021). These issues are pervasive across domains where RL is employed, but are particularly profound in military applications where the complexity of the environment necessitates continuous learning, skill composition, and rapid adaptation to new information (Hadsell, 2019). For an extensive review on general RL challenges, Dulac-Arnold et al. (2021) provide a relatively recent and comprehensive work.

The remainder of this section discusses four challenges that are specifically associated with developing and deploying RL-based DSSs in a defense and security context. The four challenges discussed are: complex scenarios and operations; availability of data; connecting with simulators; and connecting with decision makers.

#### Challenge 1: Complex scenarios and operations

**Figure:**
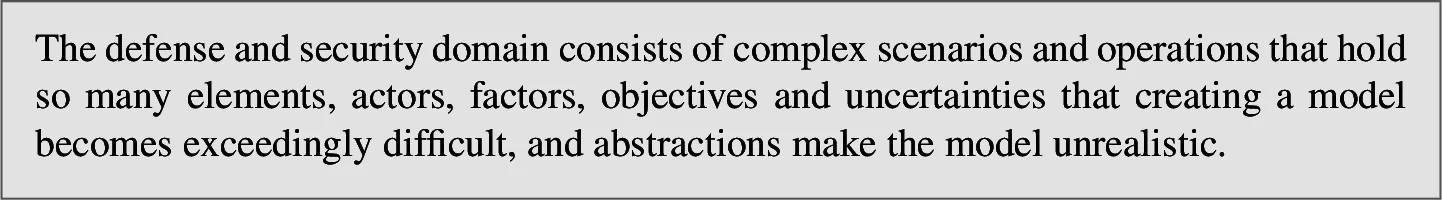

Many decision problems that may benefit from decision support, be it RL-based or not, within a defense and security context are complex due to a wide variety of factors. Although not all factors are relevant in every situation, factors include: multiple actors,^25^ multiple conflicting objectives, either for a single actor or between different actors,^26^ a range of uncertainties,^27^ and non-stationary environments.^28^ Given this reality, a significant challenge towards the deployment of RL-based DSSs is the ability to strike a balance between modeling the features of a decision problem accurately enough to provide meaningful decision support, but abstract enough such that the resulting sequential decision model and its implementation as a simulation is not too cumbersome (Weyland et al., 2024).

As an example of a complex military decision problem, consider the recent conflict between Hamas and Israel. Although originally limited primarily to these two actors, the conflict spread to two fronts—in Gaza between Hamas and Israel and in Lebanon between Hezbollah and Israel—as well as engagements between Israel and other actors including Yemen’s Houthi rebels and Iran. In addition, other non-state actors play a role, such as the United Nations Relief and Work Agency for Palestine Refugees in the Near East. Beyond operations, scenarios considered within a nation’s defense planning share similar complexities, such as (Harrison et al., 2020): multiple objectives due to the breadth of military operations considered; multiple actors, such as Air Force, Army, and Navy; and a non-stationary environment due to changes in the geopolitical landscape, defense policy, and adversary’s capabilities. Designing models, and implementing the corresponding simulations, that capture the key features and their interdependencies in such operations and scenarios is not straightforward (Davis, 2003, p. 257). Taylor et al. (2009) show that there exists a lack of real-world involvement in simulation modeling research and an even greater lack of evidence of real-world benefit for many domains, including the defense domain.

Modeling sequential decision problems that involve multiple actors, either cooperating, competing or both, inherently encourages a multi-agent approach.^29^ The main general challenges of MARL, among others, are scalability, non-stationarity and partial observability (Huh & Mohapatra, 2023; Zhou et al., 2023). Canese et al. (2021) argue and visualize how 14 MARL algorithms are impacted differently by these challenges. On top of general challenges in MARL, it has also been shown that complex dynamics in military simulations hinder MARL-based approaches (Liu et al., 2021). For example, traditional RL algorithms struggle with multi-agent dynamics in military logistics (Li et al., 2021) and high data demands may challenge training in broader scope applications (Bunch et al., 2024). Additionally, high-fidelity environments and hyper-parameter tuning make MARL-based approaches computationally prohibitive (Liu et al., 2021), especially in cooperation strategies. The computational limits call for speed-up, but faster simulation speeds may lead to unrealistic behaviors (Goecks et al., 2023).

The challenges of decision problems that involve multiple actors are compounded with the fact that each agent often has its own objectives, which may be conflicting. For the military domain, examples of such conflicting objectives are minimizing civilian casualties, maximizing mission success, while at the same time ensuring resource efficiency. Currently, the most common approach to address the challenge of multiple objectives, is to transform these into a weighted single-objective function, instead of choosing approaches from MORL. Roijers et al. (2013) however provide three distinct scenarios in which converting such a problem to a single-objective one is impossible, infeasible, or undesirable. Designing the objective function is an essential and non-trivial task, as the RL agent may produce negative side-effects or hack the reward function to optimize the score (Amodei et al., 2016). A great example of both can be seen when an RL agent learns to play hide and seek (Baker et al., 2019). Designing an (possibly multi-)objective function is a challenge that is not unique to the defense and security sector, except that the quality and quantity of available data for designing and testing a well-working objective function, single or multi-objective, is even more challenging than in other domains (Hayes et al., 2022).

There exists many uncertainties in defense and security scenarios and operations that may impact a decision (see Table 1) and which are often not included in the model to make the model manageable. Table 7 highlights that only a limited number of uncertainties are addressed in the surveyed defense and security models. Not accounting for such uncertainties may lead to a *Sim2Real* gap, in which a learned decision policy works great in the simulator but not in the real world (Höfer et al., 2021). Adding a multitude of uncertainties may improve the reliability of the results but may also negatively affect RL training through instability and can simultaneously hinder the interpretability or reproducibility. This has created the sub-field of *Robust Reinforcement Learning* (Moos et al., 2022) and *Uncertainty Aware Deep Reinforcement Learning* (Lockwood & Si, 2022).

The defense and security environment is inherently non-stationary, such as when new actors appear or the objectives of actors shift. Taking non-stationarity into account is complex, as changes in the environment usually cannot be predicted in advance. Research typically ignores this completely and simply creates a model for the current environment dynamics. We not only see a lack of taking non-stationarity into account for defense and security applications, but we also see a lack of research that addresses this topic. For existing work on this topic for other domains see Chandak (2022); Chen et al. (2021); Liu et al. (2024); Feng et al. (2022).

**We recommend** that creators of RL-based DSSs for the defense and security domain spend sufficient time and effort on designing a sequential decision model that, if required, adequately addresses multiple actors and objectives, a range of uncertainties, and non-stationary environments, as this model forms the backbone of the system. We mention three main activities that support in designing such a model.

(1) System modeling is a profession and may be regarded as an art form. There exists a plethora of methodologies that provide support in creating accurate models, and we would like to point to Model-Based Systems Engineering (MBSE) (Estefan, 2007) as a starting point for the reader.

(2) We furthermore recommend to critically think about the decision support question at hand. A model does not have to include every aspect of the decision problem, as long as it is useful to the decision maker. In this sense there is a big difference between decision support and automation, as for decision support there is always a human-in-the-loop. The realism of the simulations is often referred to as fidelity, and there exists research that tries to find a well-working fidelity for a decision support question (Weyland et al., 2024; Truong et al., 2022). In particular, we encourage to reflect critically during the modeling process, whether a basic setting with a single actor or objective is chosen based on convenience or necessity. Given the current state of development these kinds of simplifications are unnecessary and can reduce the practical value in supporting decisions significantly.

(3) For ensuring that the created model, the learned policy, and the DSS are adequate, verification and validation activities should be performed early and regularly. By doing so, design choices of the sequential decision model can be tested and improved upon early in the development. Myllyaho et al. (2021) and Landers and Doryab (2023) provide an overview of validation methods for AI systems in general and available toolkits should be used as a starting point (Dreossi et al., 2019).

#### Challenge 2: Availability of data

**Figure:**
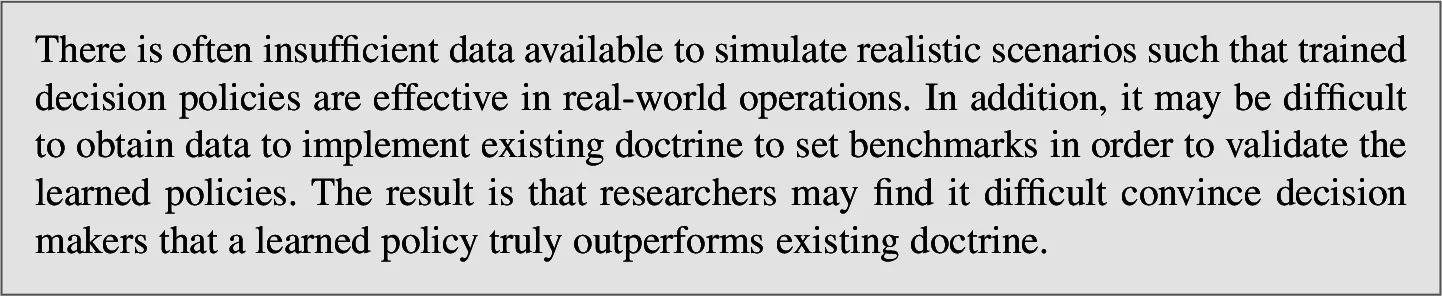

When using RL to learn a decision policy, the need for quality data is a persistent issue. For example, given the unified framework for sequential decisions in Figure 6:

- when modeling a *sequential decision problem*, the choice of elements in the model is strongly restricted by the need to have data available for those elements, i.e., data may have too much or too little detail;
- similarly, quantifying the uncertainties in the resulting *sequential decision model* may require data from a variety of sources at different classification levels, which may not be possible to obtain; and
- when designing *decision policies* and benchmarks, their structure, parametrization, and constraints may require access to both existing doctrine and outcomes of its use.

As an example of the need for data, consider the portfolio optimization problem that resides within defense planning. When conducting defense planning, many nations develop a 20-year plan for their capability investments. To do so, data must be collected on existing capabilities, potential projects (including costs, benefits, schedule), budgets, future scenarios defined in policy, and both allies and adversaries projected capabilities. Much of this data includes significant uncertainty, is based on assessments by subject matter experts, and is non-stationary due to changes in the defense and security environment (Fitzsimmons, 2006). The issue of data scarcity and the use of subject matter expertise in the context of defense planning was highlighted in the NATO report entitled *Handbook of long term defence planning* (Bakken, 2003).

Not only in defence planning, but across the defense and security spectrum it is well-acknowledged that accessible, documented, and relevant data is scarce (Svenmarck et al., 2018; Schadd et al., 2022a; Rettore et al., 2024; Vasankari & Saastamoinen, 2024). This represents a significant challenge to train an RL agent as these data are used to specify the initial state $S_0$, including “information about initial estimates (or priors) of parameters, as well as assumptions about probability distributions and functions” (Powell, 2022, p. 560) and to introduce exogenous information $W_{t + 1}$ after decisions are made. When available, historical data is often used for these purposes; however, such data can be noisy and due to the complexity and dynamics of military operations (Schadd et al., 2022a) and has been identified as a source of fog limiting a commander’s ability to achieve accurate situational awareness, and thus make good decisions (Summers et al., 2005). As mentioned, subject matter experts may be used to provide or augment available data, although this introduces its own set of concerns such as the use of heuristics and biases when providing estimates (Williams, 2010). For example, an officer who has witnessed several IED attacks may overestimate the probability of future IED attacks occurring as a result of the availability heuristic (Van Den Bosch & Bronkhorst, 2018). Furthermore, when considering future operations that include new equipment, doctrine, etc., data may not be available or only partially available. In this case, a large portion of the initial state and exogenous information may consist of assumptions and hypotheses, further complicating matters when comparing a learned policy to a benchmark.

When data is available, it is often collected through several heterogeneous sources, including sensors, imagery, lessons learned, war games, exercises, etc. (Kostopoulos, 2019). For example, Doll et al. (2021) combined data from Open Street Map, the Shuttle Radar Topography Mission, and user input to train an RL agent in their ReLeGSim environment. Likewise, Rempel and Shiell (2023) used data collected from the Canadian Coast Guard, Royal Canadian Air Force, and civilian medical community to train an RL agent to recommend which individuals to transport from an evacuation site based on their assigned triage category. In both instances the data sources were siloed, which has a negative impact on the development of AI (Majzoub, 2024), including RL-based DSSs. This negative impact includes increased time collecting and cleaning data, as well as the potential to unintentionally ignore valuable data.

Two additional, yet related issues, complicate the data requirements for applying RL for decision support. The first being that environments are non-stationary; and the second that when deployed, the RL-based DSS will be used in environments not encountered during training. In both cases, as stated in the previous challenge, significant changes in the environment cannot usually be predicted in advance (see sources previously mentioned in Subsubsection 5.2.1). In addition, in the latter case, depending on the length of time since training or system development, the environment may have evolved drastically.

**We recommend** that in order meet the challenge of data availability it is necessary that there is a structured approach to data collection during exercises, war games, and other training activities. We believe that due to data scarcity it is crucial that allied nations share their data and that there is a unified single common structure/format to enable seamless data sharing. Google recently proposed RLDS (Ramos et al., 2021), which provides a standard format of RL datasets in order to easily share data (in this case on TensorFlow (Paper, 2021)) without any loss of information and to agnostic of the underlying original format. Such shared data may be used for validation, or for learning a policy offline. Two community standards that focus on sharing data for offline learning are RLUnplugged (Gulcehre et al., 2020) and D4RL (Fu et al., 2020).

A good step towards leveraging data sharing initiatives within the military context is the Alliance Data Sharing Ecosystem (NATO, 2024c): the “availability of AI-ready, quality data is a prerequisite for NATO to maintain its technological edge and military effectiveness”. A good example of where integrated data sharing has started to happen is in the new Combined Joint All-Domain Command Control (CJADC2) system in the US (CDAO, 2024). This initiative, run by the US Chief Digital and Artificial Intelligence Office, has the goal of aiding the modern Joint Force Commander in achieving decision advantage by providing informed decisions with speed and accuracy. The CJADC2 is a set of connected capabilities providing the Joint commander with sensors and systems information across tactical, operational, and strategic levels. Crucial to our recommendations CJADC2 also connects the command and control systems of key US allies and partners thereby enhancing data sharing capability.

As a further step towards using standard environmental platforms and having shared data, we recommend the development of a “wargaming cloud" for joint operational wargames and exercises. This would also facilitate data sharing and collaboration among military nations, enhancing strategic analysis and adaptability (Dasgupta, 2021). This recommendation also connects to the US Chief Digital and Artificial Intelligence Office initiative, “Global Information Dominance Experiments" (GIDE). These experiments have the goal of supporting the CJADC2 and combine the efforts of international allies and partners to demonstrate how to use iterated experimentation to mature data and software capabilities (CDAO, 2024).

Lastly, given that the real-world defense and security environment is dynamic and may differ significantly from that in which an RL agent was trained, it is expected that a *continual RL* approach (Abel et al., 2023) will be required. To enable this approach, RL-based DSSs will need to be connected to either live data sources or have their databases systematically updated on a regular basis.

#### Challenge 3: Connecting with simulators

**Figure:**
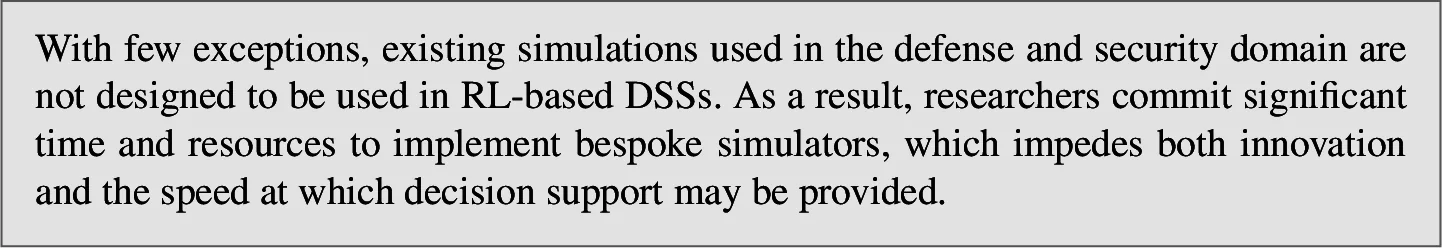

Simulators are used extensively within the defense and security domain for the purposes of decision support. However, two key issues exist when seeking to use a simulator in an RL-based DSS: first, the lack of availability of an applicable simulator that models the sequential decision problem to be solved; and second, if a simulator is available, in general it lacks support to interact with an RL agent.

The first issue stems from the lack of available appropriate simulators for military use. While simulation and modeling are widely used in the military, the increasing complexity of warfighting and opportunities of modern computational capacity have challenged monolithic, low fidelity simulators (Hofmann, 2004). Typically, such simulators are designed to address a specific problem and are not generalizable to other problems even within the same context. While these narrow simulators can be integrated into a system of systems to provide decision support to a new, more complex setting (Hofmann, 2004), such integration is not trivial. As a result, researchers tend to either use existing simulators, accepting their shortcomings, or put extensive effort into designing, developing and implementing a new simulator for the research task (as evidenced in Table 5). This hinders the ability to implement RL-based DSSs, not only due to the majority of effort being required to construct the simulator, but also due to challenges with designing effective operator interfaces (Goecks et al., 2023) and that software bugs in simulators can direct RL agents to incorrect solutions (Baker et al., 2019).

Regarding the second issue, it has been acknowledged that “most military simulations do not have APIs [Application Programming Interfaces] that allow reinforcement learning algorithms to connect to the underlying program” (Boron, 2020, p. 19). This lack of connectivity is due in part to that developers of military simulations and RL exist in distinct communities, each have their own focus, i.e., the military simulation community tend to focus on mission analysis, acquisition, and campaign models (Hill & Miller, 2017; Song et al., 2015), whereas the RL community focusses on research to support the engineering of algorithms (Towers et al., 2024). In practice, this separation is further highlighted by the fact that environments, also known as gyms, built in the RL community are usually written in Python, whereas constructive simulators are usually written in different languages (Briggs, 2024). Furthermore, as pointed out by Towers et al. (2024), the RL community itself suffers from a lack of standardization in the implementation of environments (as well as algorithms). The result is that due to different requirements and a lack of standardization for both simulations and RL agents, it is a challenge to gain full access to either.

Fortunately, recent research has attempted to rectify this issue. In particular, Hua (2023) developed an interface between the *Command: Modern Operations* simulator and RL agents. While this simulator is widely regarded as the state-of-the-art simulator for military operations and is capable of handling a wide range of engagements, the professional license required comes with a considerable cost, downplaying its wider applicability and research opportunities. Likewise, RAND has recently developed software that “enables the use of state-of-the-art reinforcement learning (RL) methods in many existing constructive simulations without requiring significant additional coding” (Briggs, 2024, p. 1). The software, described as a *harness*, is designed to wrap around a constructive simulation and control its execution. While this is promising, the author demonstrated their approach using only two simulators, the *Advanced Framework for Simulation, Integration, and Modeling* and RAND’s *Target Accessibility Model*. Thus, the wider applicability of such a harness remains to be seen.

**We recommend** that to address the problem of having an appropriate simulation environment, that there is the construction of a standardized military environment platform that allows sharing and integration of simulators across allied nations. The platform would enable far-reaching use of simulators for RL solutions while standardization would guarantee that RL integrability is assured with minimal overhead and redevelopment. Apart from providing synergies and sharing of resources, it should allow the growth of complex environments by bringing together independently developed simulators. The challenge where simulators are not chosen for their use in RL (but for their use in human training) could then be overcome as the unified platform would be developed specifically with the required speed and appropriate interface to support training RL agents.

There are few community examples from the military and security domain that demonstrate this idea and are suitable for decision support, B-ACE for air combat multi-agent scenarios (Kuroswiski et al., 2024) is one example that could be used for decision support. For control tasks, the Tunnel simulator (Search, 2025) is shared with the community.

We have also observed the creation of such simulation environments for other domains. Xu et al. (2024) introduces OmniDrones, an RL environment for learning in drone control. It allows users to design and experiment with various application scenarios, including benchmark tasks. Chan et al. (2024) provide an overview of 28 drone control simulators useable for RL. Microsofts CyberBattleGym (Team, 2021) is an experimentation research platform to investigate the interaction of automated agents operating in a simulated abstract enterprise network environment. The simulation provides a high-level abstraction of computer networks and cyber security concepts. Its Python-based Open AI Gym interface (Towers et al., 2024) allows for the training of RL agents. Another example is Google’s Brax engine (Freeman et al., 2021). Brax is a differentiable physics engine for research and development of robotics, human perception, materials science, RL, and other simulation-heavy applications. These three examples demonstrate how appropriate simulation environments can reduce the development efforts for RL in their respective domains.

#### Challenge 4: Connecting with decision makers

**Figure:**
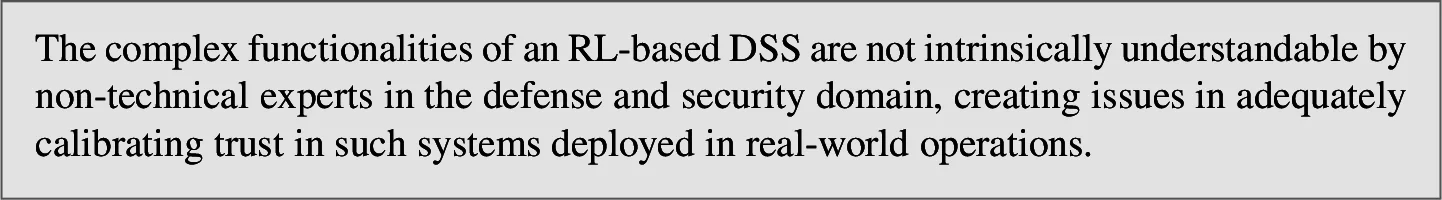

While using RL in DSSs has been researched in areas such as manufacturing processes (He et al., 2021), logistics and transportation (Li & Wu, 2022; Rabe & Dross, 2015), and medical care optimization (Liu et al., 2020), public research in the military domain is scarce. Even the verification and validation of (deep) RL decision policies that display tactical behavior is a challenging task and a novel field of research (Landers & Doryab, 2023). In these systems, a core challenge for the decision maker is to understand the system well enough to balance trust and skepticism towards its suggested decisions. Trust in DSSs is essential for military users that are faced with critical decisions. A practical example, albeit not RL based, can be seen with the Patriot system and its fratricides, where own fighter jets were fired upon due to wrong recommendation from the system (Hawley, 2007); the operators and commanding officers did not have enough time or reasonable ground to question the system’s performance in these particular cases.

It has been demonstrated that RL agents that employ a policy based on deep neural networks face reproducibility challenges during training. Policies trained in a test bed cannot easily be re-trained with the same policy as a result. A reason for this is the non-deterministic nature of environments and inherent variance (Henderson et al., 2018). These issues might already raise skepticism about the quality of suggested decisions. This tendency can be increased if the suggested decision is not relatable to the human expert. Classically, this has been observed in many board games including Go (Metz, 2016), where long established ideas were often shown incorrect by trained RL agents. Utilizing RL for larger processes could decrease trust in the system as interpretability decreases with application size (Bunch et al., 2024).

Trust calibration refers to adjusting human trust to the performance and reliability of systems, such as robotic systems (Perkins et al., 2021). The same applies to RL-based DSSs. As an adversarial example, impressive RL feats such as AlphaStar and OpenAI Five supremacy in StarCraft II and Dota 2 games respectively, were partially due to how human players had not encountered such opponents before (Gault, 2018; Jeffrey, 2019). Once human players became acquainted with the tactics employed by the RL trained opponent, i.e., calibrated to their performance, weak points could be efficiently exploited. In other words, calibration was required on the part of human players to understand the solutions of the learned policy. Likewise, it can be argued that trust calibration is required for the human and DSS to cooperate effectively.

While explainability is vital, it remains a general challenge for AI methods (Dwivedi et al., 2023). As shown in Table 5, the explainability attributes of used methods are neither thorough nor robust. Applications are narrow and focused, conflating explainability and trust to some extent. Post-hoc and statistical explanation methods are less useful because users require immediate access to the reasons behind the recommendations of the RL-based DSS, demonstrating again that the objective needs to be true explainability as opposed to interpretability.

This distinction can be also viewed from the perspective of non-technical experts using systems with technical solutions, where we have to divide system understanding into technical and functional (Ginzberg & Stohr, 1982). According to Ginzberg and Stohr (1982) the usage and process can be seen as functional perspectives, while technical understanding of an RL-based DSS can be defined as knowledge and understanding of the technological components. As stated by Huntington (1963), in the military the need for specialized (technical) expertise conflicts with the need for experienced generalists as managers and commanders.

Lastly, the overall human-computer interaction in AI and DSSs has been proposed to be enhanced with Large Language Models (LLMs) (Rajashekar et al., 2024). As explainability entails interaction between the RL agent and non-expert users, LLM could be leveraged within an interface that enhances explainability. However, this raises additional issues, as LLMs have their own explainability challenges (Cambria et al., 2024), are generally neither fine-tuned for military use (Ruiz & Sell, 2024) nor protected against possible back door attacks (Zhao et al., 2024), which would make their use in critical systems problematic.

**We recommend** a strong investment in explainability methods for the defense and security domain including those explored in Subsection 2.5. Specifically one would want an explainable policy dissection so that the RL-based DSS can provide recommendations accompanied by reasons that pertain to the environmental state in real time. This is currently being developed for RL cyber defense agents (Frazer-Nash, 2024) and should be extended to other military areas to make RL-based DSSs both relatable and allow trust calibration.

To support a stronger connection with decision makers, the field should offer a background for developing understanding and trust in the systems and their possibilities for future users (for a recent example of this, see Vereschak et al. (2024)). In addition, non-technical experts should be brought into the RL-based DSS development process as early as possible. Likewise, education and training are recommended for providing audience-appropriate functional understanding of advantages and shortcomings of technological model components. If LLMs are to be used within an RL-based DSS interface, they need to be fine-tuned for the domain, secure for defense purposes, and comply with ethical guidelines for trustworthy AI.

To overcome the lack of empirical validation of the trained decision policies, future assessments could be anchored in established TEVV frameworks, which provide structured criteria for assessing system readiness across simulation, controlled trials, and operational environments. Applying such a framework would make it possible to distinguish between early-stage conceptual contributions and mature, decision-support-ready systems. As a concrete recommendation, we suggest adopting minimal evaluation standards for (1) the models and simulators used for RL, (2) the policy that has been trained, and (3) the decision support system in its entirety. This includes: (i) comparison against established baselines or heuristics to ensure added value, (ii) sensitivity analyses to capture robustness under varying assumptions and environmental conditions, and (iii) transparent reporting of limitations. Establishing these practices would both improve reproducibility and accelerate the translation of RL-enabled DSS into operationally relevant settings. While there is a broader need for a universal checklist of explainability requirements that could standardize such practices across domains, developing and defending such a checklist lies beyond the scope of the present paper.

Lastly, it is recommended that RL-based DSSs be used as part of wargames (Black & Darken, 2024) and training exercises. The experience of using an RL-based DSS in a wargame context can both aid its design and be useful in determining requirements for effective explainability to non-experts. Likewise, utilizing RL-based DSSs in wargaming introduces current and future decision makers to the system, fostering understanding of its strengths and weaknesses, feeding innovation, and developing trust. This is crucial for establishing the link to end-users and developing appropriate trust. Thus, using an RL-based DSS in a training context offers a low-risk method of developing trust and facilitating development of explainable systems.

## Conclusion

In this article, we presented a review of applications of RL for decision support within a defense and security context. In particular, we focused on articles from those nations participating in the NATO SAS-181 RTG, that were peer-reviewed, and whose primary aim is to provide decision support.

In our methodology we searched various online sources and national databases for relevant work. Only when an article addressed all three criteria (i.e., RL, decision support, and defense and security context) it was deemed in scope of this review. This excluded a large part of research on using RL for the automation of tasks. Furthermore, only research published from the year 2000 onward was in scope. After this extensive search, we found 20 articles to be in scope of this review.

To gain a high-level overview of the research field, we chose to describe each article with 19 characteristics. The chosen characteristics are specific for the domain and demarcate the discovered works. We encourage readers to re-use the presented characteristics for reviewing other research areas. For attributing a characteristic to an article, at least three authors of different countries made independent assessments and discussed any differences plenary with all co-authors. This resulted in the overview as presented in Table 5 which formed the basis for further analysis.

We observed several different application areas for RL-based decision support in the defense and security domain. While many concern the tactical engagement of forces (i.e., force employment), others address concept development, maintenance scheduling, and investment planning. The land, air and naval domain are well-represented, but cyber, space and MDO are underrepresented. Across these domains, all research reviewed assume a static environment that does not change during the simulations, and we recognize the need for the dynamics of an environment to be taken into account in order to obtain a policy that is robust to the wide range of uncertainties present in the defense and security domain. Yet, many articles took exogenous uncertainty into account to some extent, often by modeling the unknown adversary behavior in some manner. The current approach within the research domain also lacks the means for successful validation of the trained policies, with only few efforts being made to show the adequacy of the learned policy in the real world. Furthermore, explanations on why a policy prefers one action over another are rarely generated. Typically the research explained a policy’s behavior in the author’s own words, but no inherent methods to automatically justify a decision are presented. Finally, we conjecture that these aspects may contribute to that all applications of RL-based DSSs in the defense and security context are still in the theoretical or proof-of-concept stage.

Finally, by using the gained overview of the field of RL-based DSSs for the defense and security domain, we have formulated four challenges that hinder the progress of researchers. For each challenge, a possible way forward has been formulated. (1) The first challenge is the degree of difficulty to create a model of complex defense and security scenarios and operations. We recommend to invest in a system modeling approach, to critically weigh what to include and exclude in the simulation model, and to account for verification and validation activities. (2) There typically is not sufficient data for creating models, quantifying uncertainties, or creating benchmarks. We believe the way forward is to invest in data collecting, curating and sharing activities, and to develop a “wargaming cloud” for joint operational wargames and exercises. (3) The overwhelming majority of existing simulators are not designed to be used in RL-based DSSs, leading to the creation of bespoke simulators by researchers. We recommend to investigate the possibility of creating a standardized military simulation environment that allows the sharing of simulators and creates synergies between existing simulators. (4) There currently is insufficient attention paid to how users interact with a trained RL agent and its learned decision policy in a DSS. We recommend a strong investment in explainability methods and to involve users early and frequently, possibly already by using the DSS during wargames and training exercises.

To conclude, we have observed that most efforts of RL-based DSSs for the defense and security domain remain in the low TRL levels. Military decision-making use cases span from real-time to decades-ahead planning, and must address partial unknowns and a plethora of uncertainties. In spite of high stakes of failure, RL emerges as a promising approach for adaptive, robust, and efficient solutions, assuming that the challenges are overcome with further research and advances in technology. A standard way of working within this application domain has yet to form. We believe it beneficial that a general framework of thought should be created that can guide researchers and developers when working on RL-based DSSs in the defense and security domain—specifically because the DSS is a much larger system compared to the RL model itself. Proposing such a framework of thought is future work of the authors.

## Footnotes

1 The SAS Panel “conducts studies and analyses to enhance decision-making in matters of strategy, capability development, and operational activities within NATO, NATO Nations, and partner nations” (NATO STO, 2024a, p. 24).

2 For example, the authors state that a “software agent receives keystrokes, file contents, and network packets as sensory input and acts on the environment by displaying on the screen, writing files, and sending network packets” (Russell and Norvig, 2016, p. 34).

3 NATO defines the term *doctrine* as “fundamental principles by which military forces guide their actions in support of objectives. It is authoritative, but requires judgment in application” (NATO Military Committee Joint Standardization Board (MCJSB), 1973). In essence, the determined doctrine defines the boundaries and guidance of available actions.

4 NATO defines the term *decision* as “a determination of future actions” (NATO Digital Policy Committee (DPC), 2005).

5 In the military, tactical is seen as the lowest level with the shortest temporal outlook and a narrow scope (Greener, 2009). In other fields, these qualities are attributed to the operational level.

6 To underline the compatibility of the OODA-loop framework and MDMP, the APP-28 planning process used by NATO showcases in its Annex B (NATO, 2019) a comparison table with several different MDMPs. In the comparison table, the process phases are briefly described and categorized into OODA-loop phases, portraying a strong resemblance throughout different realizations of an MDMP.

7 Wargames are analytic games that simulate aspects of warfare at the tactical, operational, or strategic level. They are used to examine warfighting concepts, train and educate commanders and analysts, explore scenarios, and assess how force planning and posture choices affect campaign outcomes (Rand, 2024).

8 Autonomous systems are not considered to provide decision support, since these systems are making decisions themselves (Kalff et al., 2024).

9 The five classifications are: communication-driven DSS, data-driven DSS, document-driven DSS, knowledge-driven DSS, and model-driven DSS.

10 Regret up to decision epoch *T* is defined as the accumulated difference between the reward of an optimal policy and that of the learning policy: $\textrm{Regret}(T)=\sum _{t=0}^{T}(r^\star _t-r_t)$, where $r^\star _t$ and $r_t$ denote rewards under the optimal and learned policies, respectively. See also Burnetas and Katehakis (1997).

11 In the universal modeling framework, the contribution function $C(S_t, X^\pi (S_t))$ may be deterministic given the state and decision, or stochastic through its dependence on exogenous information and state transitions; this formulation subsumes the standard MDP reward definitions commonly written as $R(S_t, A_t)$ or $R(S_t, A_t, S_{t+1})$.

12 Reward indexing follows Powell’s formulation, in which contributions are associated with the state–decision pair at epoch *t*, rather than the $R_{t+1}$ convention used in some RL texts.

13 For example, the Centralized Training with Decentralized Execution (CTDE) paradigm allows agents to train with a global state and only adds local restrictions during execution. In military decision support this occurs, when centralized information (e.g., from surveillance or intelligence systems) may be available during planning and training, but in the field, units must act autonomously with only partial local observations.

14 Note that many authors use the term *cost function approximation* or *cost-to-go* to refer to a value function approximation (Powell, 2016, p. 328).

15 Possibly changing the simulation models structure, level of detail, or simply fixing bugs.

16 For example: the characteristic *Simulation Horizon* is associated with *model first*, and specifically the *modeling* step itself; *Policy Category* is associated with *model first* as well, however maps to the *decision policy design* step; and *Type of Simulator* strongly influences how a given problem is solved, and best matches to the *Algorithm strategy selection* step since it directly affects the selection of RL algorithm.

17 Force employment is the ultimate purpose of a military force and is enabled by both Force Generation and Force Projection. Force Employment is how a military force is used to achieve the desired effects or influence on the operating environment. A military force does this by directly or indirectly engaging other actors (enemy, friendly, or neutral) and sustaining their forces being employed. Force Projection is how a military force is moved from where it is generated to where it will be employed. A military force does this by moving its forces from those positions (forward-deployed, staging bases, ports, installations) it has established to project and rapidly employ the forces. Finally, Force Generation is how a military force is created from available resources and personnel, with the aim to be projected and employed. A military force does this by preparing the forces it has developed according to its strategies and operational requirements that describe and direct the desired military effects and influences that they aim to create in the operating environment.

18 Many military forces within the NATO ecosystem have joint military capabilities. In the post-World War II restructuring of military forces, the emergence of integrated command structures that cut through inter-service rivalries was common throughout the militaries of NATO members. These joint capabilities are associated with a collective focus and cooperation between traditional military services (think of United States Joint Chiefs of Staff or the United Kingdom’s Permanent Joint Headquarters). However, joint command structures promote coordination with other armed services, while a multi-domain mindset must go beyond that. The NATO definition is the orchestration of military activities, across all domains and environments, synchronized with non-military activities, to enable the Alliance to deliver converging effects at the speed of relevance (NATO, 2022, 2023). As joint mainly concerns coordination of military services, we prefer MDO for this characteristic.

19 For example, Zhang and Yu (2020) provides a structure of typical and popular algorithms into model-based, model-free, value-based, or policy-based. Almahamid and Grolinger (2021) provides a structure based on the number of states and actions. Shakya et al. (2023) provides a summary of model-free and model-based deep RL algorithms, and a view based on value optimization or policy optimization.

20 Eligible values include those listed in Bertsekas and Tsitsiklis (1996), Sutton and Barto (2018), Powell (2011), Powell (2022).

21 A special case is the i2Sim simulator (Marti et al., 2008). Although it has been used in a wide variety of studies, we classify it as bespoke. It is not open source. It is from academia, not purchasable from a commercial or government institution.

22 Such as the innovation readiness levels (Lee et al., 2011) and science readiness levels (European Space Research and Technology Centre (ESTEC), 2015), with the most-used one being the Technology Readiness Levels (TRLs) (Mankins, 1995).

23 An approximate mapping to the TRL levels is as follows—TRL levels 1–3: theoretical, TRL 4–5: proof of concept, TRL 6–7: field tested and TRL 8–9: operational.

24 If it all possible, this explanation should be a human-grounded explanation, one that approximates the reasons which human agents provide and receive whenever justifying decision making. This will ensure the output of the system is perceived (justifiably) as trustworthy. The theory of human-AI epistemic quasi-partnerships (EQP) provides a framework for articulating conditions for human-grounded reasons (see Hase and Bansal (2020) for a review). A recent example of explanation capability is given in Saldiran et al. (2024), which propose one local and three global explanation methods for a policy that learned close air combat.

25 Such as branches of a nation’s armed forces, nations, international organizations, and militias.

26 Such as maximizing operational readiness of vehicles while minimizing the costs.

27 Such as the weather, hardware malfunctioning, human behavior or partial observability of the environment which complicated the decision-making environment (Vasankari & Virtanen, 2025).

28 Such as changes in tactics and material of the adversary or alternated logistic due to shifting demands, disruptions from attacks, or changes in operational priorities (Gachet & Haettenschwiler, 2003). Non-stationarity demands continual adaptation through ongoing learning (Asher & Barton, 2018).

29 Having multiple actors in the scenario does not necessarily mean a multi-agent approach. For example, when an opposing force or third party is controlled by a simple rule-based behavioral model, it can be regarded as part of the environment as it will react in a deterministic and predictable manner to the actions of the agent. The problem becomes multi-agent when there are multiple agents learning a policy at the same time, and these agents may belong to the same faction. For example agents that represent trucks, warehouses, and supply officers, coordinating to ensure efficient transport and storage while avoiding bottlenecks. Defense and security scenarios typically contain many such actors that may be modeled with a learning agent.
